# Electroacupuncture combined with cognitive rehabilitation outperforms cognitive rehabilitation alone in treating post-stroke cognitive impairment: a randomized controlled trial

**DOI:** 10.3389/fneur.2025.1507475

**Published:** 2025-01-29

**Authors:** Yisha Guo, Tingting Sun, Fengxi Qiu, Xueyi Li, Weiwei Cui, Zhenhua Liao, Jiajia Yao

**Affiliations:** Yangzhi Rehabilitation Hospital Affiliated to Tongji University (Shanghai Sunshine Rehabilitation Center), Shanghai, China

**Keywords:** cognition, DTI, electroacupuncture, stroke, network analysis, graph theory

## Abstract

This study aimed to evaluate the effects of a 12-week intervention combining electroacupuncture (EA) with conventional cognitive rehabilitation (CR) on cognitive recovery and brain network topological properties in stroke patients. Thirty-four post-stroke cognitive impairment (PSCI) patients were randomly assigned to either an EA + CR group or a CR-only group, with both undergoing five weekly interventions for 12 weeks. Cognitive assessments and Diffusion Tensor Imaging tests were performed pre- and post-intervention. Primary outcomes included the Montreal Cognitive Assessment (MoCA), Digit Span Test (DST), Auditory Verbal Learning Test (AVLT-H), and Aphasia Screening Scale. Secondary outcomes measured brain network global and nodal properties. The EA + CR group showed significant improvements in MoCA (*p* < 0.001), AVLT-H (*p* = 0.004), and in areas like naming (*p* = 0.022), attention (*p* < 0.001), and delayed recall (*p* < 0.001). Local brain network metrics, such as clustering coefficients (Cp) and local efficiency (Eloc), were significantly higher (*p* < 0.05) in the EA + CR group, though global efficiency (Eglob) was unchanged. Improvements in Eloc were positively correlated with MoCA scores (*r* = 0.0716, *p* = 0.018). Specific brain regions, including the medial and paracentral cingulate gyrus, inferior temporal gyrus, and left supramarginal gyrus, exhibited significant nodal property differences, suggesting that EA may enhance cognitive recovery by targeting cognitively relevant regions. These findings indicate that EA, combined with CR, is a safe and effective treatment for cognitive impairment following stroke, potentially via structural brain network improvements.

**Clinical trial registration:**

https://clinicaltrials.gov/, ChiCTR2200066160.

## Introduction

Post-stroke cognitive impairment (PSCI) is characterized as cognitive dysfunction that manifests within 6 months following the onset of a stroke. This condition encompasses various etiologies, including subcortical ischemic infarcts, cerebral hemorrhage, and neurodegenerative disorders such as Alzheimer’s disease (AD). Approximately 30% of stroke survivors exhibit varying degrees of cognitive impairment within three to 6 months post-stroke (1). PSCI is defined as cognitive dysfunction that occurs within 6 months following the onset of a stroke.

Electroacupuncture (EA) is an adjunctive therapy that combines traditional acupuncture with modern electrotherapy, enhancing the effects of conventional cognitive training. A meta-analysis and trial sequential analysis were performed to assess the impact of adjuvant electroacupuncture on vascular mild cognitive impairment (VaMCI). The findings indicated that electroacupuncture significantly improved clinical outcomes in patients with VaMCI (2). A lot of basic research in recent years has confirmed that the cognitive function of middle cerebral ischemia/reperfusion (MCAO/R) rats was significantly improved after EA treatment (3–7). However, there is limited research concerning the clinical efficacy and underlying mechanisms of EA in the treatment of cognitive impairment following stroke.

Currently, PSCI is primarily assessed using various cognitive test scales. However, these assessments are inherently subjective and often time-consuming (8). Moreover, these assessments primarily evaluate functional status and do not adequately reflect the underlying nature of the injuries. Whether brain network property metrics can be used as imaging markers for cognitive assessment has been investigated in diseases such as AD (9), whereas the correlation between brain networks and cognitive assessment has not yet been explored in post-stroke cognitive impairment. To gain a comprehensive understanding of post-stroke cognitive dysfunction and the impact of brain network topology properties on cognitive function, it is imperative to identify a method that can objectively assess cognitive impairment in patients with PSCI. More importantly, such a method should elucidate the essential characteristics and mechanisms influencing cognitive dysfunction.

Diffusion tensor imaging (DTI) can be used to quantitatively characterize human white matter based on graph theory (10). Using this approach, previous studies have described the topological properties of brain networks under cognitive dysfunction, such as early cognitive impairment in patients with post-stroke aphasia (11, 12) and stroke after cerebral small vessel disease (SVD) (13). Brain network measures can be considered direct and independent surrogate markers of cognitive function (14, 15). However, evidence regarding changes in brain functional networks during cognitive processing in patients with post-stroke cognitive impairment (PSCI) and the impact of lesion location on cognitive function remains limited.

In this study, we examined whether EA combined with cognitive rehabilitation training offers greater benefits than cognitive rehabilitation training alone in improving PSCI. Additionally, we explored the mechanisms through which EA facilitates recovery in PSCI, focusing on changes in brain network structure.

## Methods

### Subjects

A consecutive series of patients with a confirmed diagnosis of PSCI admitted to the Department of Neurological Rehabilitation at Shanghai Yangzhi Rehabilitation Hospital (Shanghai Sunshine Rehabilitation Center) between November 2022 and July 2024 were screened for a consecutive series of patients by a consensus panel consisting of a rehabilitation therapist, a neurologist, and a radiologist. This project was approved by the Medical Ethics Committee of Shanghai Yangzhi Rehabilitation Hospital (Shanghai Sunshine Rehabilitation Centre). The ethics code is 2022–29. China Clinical Trial Registration Number is ChiCTR2200066160. Participants provided written informed consent at the time of inclusion in the trial.

Inclusion criteria: (1) age between 30 and 85 years; (2) right-handedness before stroke; (3) within 1 year of stroke onset and stable;(4) definitive stroke diagnosis: a stroke diagnosis supported by clinical or radiographic evidence; and (5) the presence of cognitive impairment: patient’s chief complaint or informant’s report or the judgment of an experienced clinician cognitive impairment following a stroke event and neuropsychological evidence confirming the presence of functional impairment in more than one cognitive domain or evidence of cognitive decline compared to pre-stroke cognition (16).

Exclusion criteria: (1) with disorders that severely affect cognition and MRI such as audio-visual abnormalities, psychiatric abnormalities, etc.; (2) cerebellar or pontine lesions or senile dementia; (3) cognitive impairment due to hypothyroidism, traumatic brain injury, AD, Parkinson’s disease, and other degenerative causes; (4) other cerebral anomalies or psychiatric disorders, or clinically significant or unstable medical conditions; and (5) use of medications that may affect the motor examination, such as antipsychotics and antiepileptics; (6) contraindications to MRI. (7) claustrophobia.

### Randomization and blinding

Participants who met the eligibility criteria were randomly assigned to either the EA + cognitive rehabilitation group (CR) or the CR group in a 1:1 ratio. The random assignment procedure was performed by a traditional Chinese medicine (TCM) physician in the Department of Neurological Rehabilitation, who did not participate in the cognitive function assessment, cognitive rehabilitation training, MR measurements, or data analysis. Various cognitive function assessments were measured by the same speech rehabilitation therapist, who did not participate in cognitive therapy and was not aware of the grouping of subjects, and electroacupuncture was performed by another TCM physician, who was not aware of the assessment results of his/her measures. Cognitive rehabilitation was performed by another speech rehabilitation therapist, who was not aware of his/her grouping and the results of his/her measurement indicators. Magnetic resonance imaging acquisition was performed by the project leader who contacted the MRI physician for testing, who was not involved in other parts of the trial. The enrolled patients were aware of the differences in the rehabilitation interventions. However, they were not explicitly told whether the rehabilitation program was an experimental or a control group, as neither the informed consent nor the verbal explanations mentioned this specific information. The main participants of the trial were blinded.

### Interventions

#### Electroacupuncture

##### Acupoint selection

PSCI in traditional Chinese medicine falls under the category of *Dai Zheng*, with the pathological location in the brain. The pathogenesis involves an insufficiency of the sea of marrow, blockage of clear orifices, and a loss of Shen (spirit) function. *Baihui* belongs to the Du meridian, located at the vertex, where the Du meridian, Triple Burner meridian (hand shaoyang), Urinary Bladder meridian (foot taiyang), Gallbladder meridian (foot shaoyang), and Liver meridian (foot jueyin) converge. It can connect the yin-yang meridians, regulate the flow of meridian qi throughout the body, restore yang energy, and harmonize yin-yang energy, making it a crucial acupoint for treating mental and emotional disorders. *Shenting* is also part of the Du meridian and is located at the Shen (spirit) region. It intersects with the Du meridian, Yangming meridian, and Taiyang meridian. According to the *Jia Yi Jing* (The Systematic Classic of Acupuncture and Moxibustion), “Shen refers to the qi of the heaven sector, and Ting refers to the courtyard.” As acupoints of the Du meridian, Baihui and Shenting have the functions of raising yang, replenishing qi, nourishing the marrow, and calming the Shen, making them frequently used in acupuncture to prevent and treat cognitive dysfunction and neurological disorders (17, 18). Modern clinical research also indicates that Baihui and Shenting are key acupoints for regulating brain function, offering significant effects in calming the spirit, regulating emotional states, and awakening the mind (19). These acupoints are of high research value for improving cognitive function. In this study, Baihui and Shenting were chosen and treated with electroacupuncture.

##### Acupoints

Baihui and Shenting.

##### Procedure

**Figure 1:**
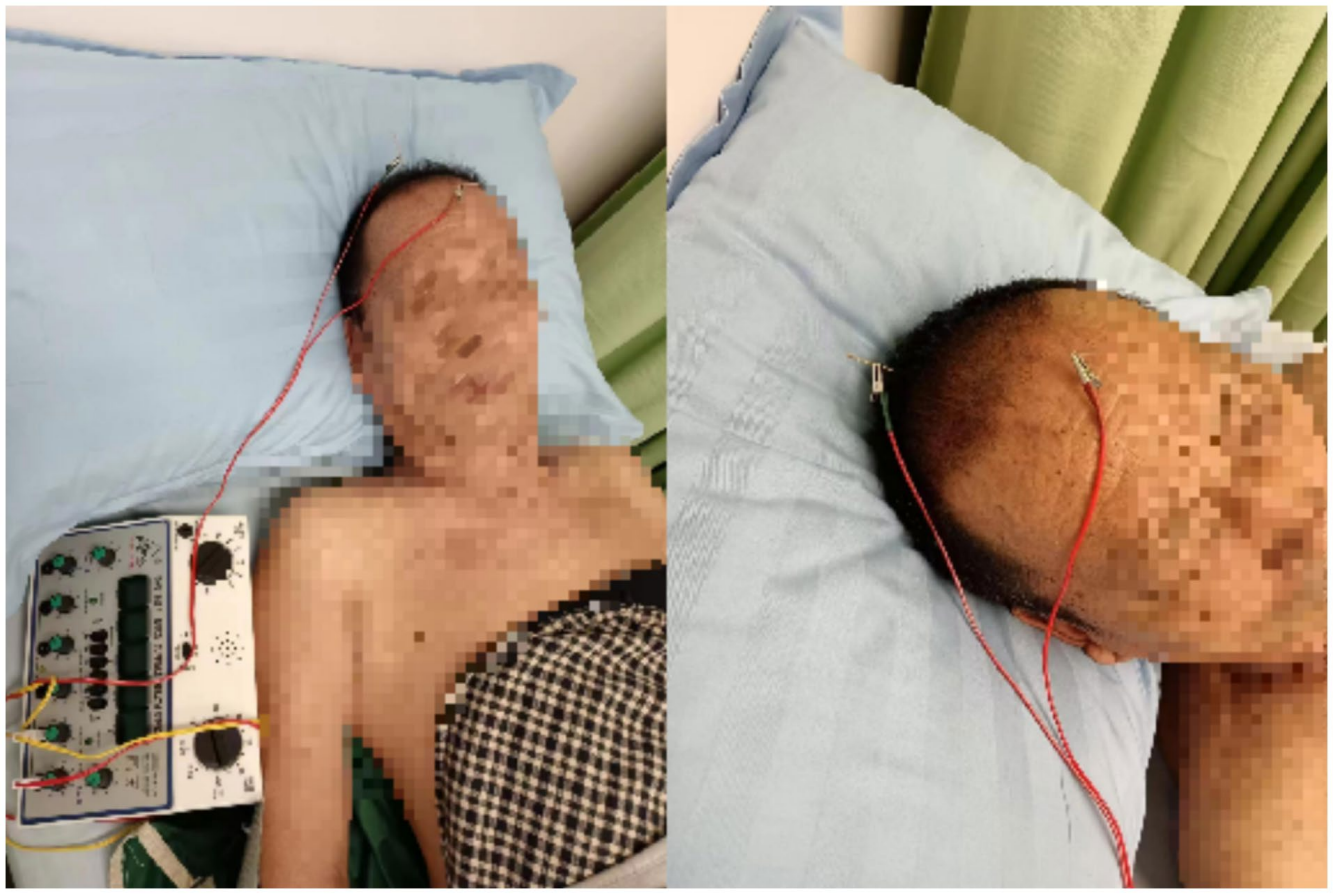
Schematic diagram of electroacupuncture operation.

The Baihui acupoint was selected. Location: at the top of the head, 5 inches directly above the midpoint of the anterior hairline, or at the midpoint of the line connecting the two auricles. The Shenting acupoint: located 0.5 inches above the anterior hairline at the midline. Acupoint selection was based on relevant literature (*Acupuncture Science*, National Planning Textbook for Higher Education in Traditional Chinese Medicine, published by China Press of Traditional Chinese Medicine) (20). The procedure was performed following the *Acupuncture Technical Operation Standard*, formulated by the China Acupuncture Association, published by the Standardization Administration of China in 2009 (21). Hua Tuo-brand stainless steel filiform needles (1.5 inches,0.35 × 0.40 mm) produced by Suzhou Medical Supplies Factory Co., Ltd. were used. After routine disinfection, the needle was inserted at an angle of about 30° to the scalp, with the direction of insertion from front to back. The needle was rapidly inserted under the scalp. When the needle tip reached the sub-aponeurotic layer, a reduction in resistance was felt under the finger, and the needle was then adjusted to be parallel to the scalp, with an insertion depth of approximately 1.5–2.0 cm. The needle was rotated at a speed of 150–200 times per minute for 1 min after insertion. The Baihui acupoint was connected to the positive electrode of the electroacupuncture device (KWD-808 I, produced by Changzhou Wujin Great Wall Medical Instrument Co., Ltd.), while the Shenting acupoint was connected to the negative electrode. See Figure 1. A sparse-dense wave frequency of 10/50 Hz was used, and the current intensity was adjusted according to the patient’s tolerance, ranging from 0.5 to 5.0 mA. The needles were retained for 30 min per session, once a day.

#### Cognitive rehabilitation training

According to the *Chinese Stroke Rehabilitation Treatment Guidelines* (22), personalized cognitive rehabilitation training should be conducted based on the patient’s cognitive assessment results. The training primarily focuses on seven cognitive domains: (1) attention and hand-eye coordination training, (2) calculation ability training, (3) writing training, (4) memory training, (5) thinking function training, (6) orientation training, and (7) language training. Training is conducted once daily, five times per week, for a duration of 12 weeks.

### Outcome measures

#### Cognitive evaluation

At both the start (week 1) and end (week 12) of the trial, a professional speech rehabilitation therapist conducted a series of standardized cognitive assessments across multiple domains. General cognitive function was evaluated using the Montreal Cognitive Assessment (MoCA) (23). Episodic memory was assessed with the Auditory Verbal Learning Test (Huashan version) (AVLT-H) (24), while short-term memory was measured using the Digit Span Test (DST) (25). Language abilities were tested using the Aphasia Screening Scale (26).

#### Imaging acquisition

Imaging was performed on a 3-T Siemens MRI scanner. Tight but comfortable foam padding was used to minimize head motion, and earplugs were used to reduce scanner noise. High-resolution sagittal T1-weighted images were acquired by a three-dimensional magnetization-prepared rapid gradient echo (t1_gre_fsp_3d_sag) sequence. The detailed scanning parameters were as follows: repetition time (TR) = 7.41 ms; echo time (TE) = 3.2 ms; inversion time (TI) =900 ms; flip angle (FA) = 9°; slice thickness = 1 mm; spacing between slices = 1 mm;176 axial slices; matrix size = 256 × 240; field of view (FOV) = 256 × 256 mm^2^; voxel size =1 × 1 × 1 mm^3^.

DTI images covering the whole brain were acquired by a single-shot echo planar imaging-based sequence with the following scan parameters: TR =3,100 ms; TE = 84.8 ms; FA = 90°; 32 diffusion-weighted directions with a b-value of 1,000 s/mm^2^, and a single image with a b-value of 0 s/mm^2^; slice thickness = 2.5 mm; no inter-slice gap; number of slices = 1,485; matrix size = 128 × 128; FOV = 256 × 256 mm^2^; voxel size =2 × 2 × 2 mm^3^.

#### Brain network analysis

A DTI diffusion scheme was used, and a total of 32 diffusion sampling directions were acquired. The b-value was 987.035 s/mm^2^. The in-plane resolution was 3.4375 mm. The slice thickness was 2.5 mm. The diffusion data were reconstructed in the MNI (Montreal Neurological Institute) space using q-space diffeomorphic reconstruction (27) to obtain the spin distribution function (28). A diffusion sampling length ratio of 1.25 was used. The output resolution in diffeomorphic reconstruction was 3.4375 mm isotropic. The restricted diffusion was quantified using restricted diffusion imaging (29). The tensor metrics were calculated using DWI with b-value lower than 1750 s/mm^2^. A deterministic fiber tracking algorithm (30) was used. A seeding region was placed at whole brain. The anisotropy threshold was 0.2. The angular threshold was 45 degrees. The step size was 0.5 mm. Tracks with length shorter than 30 or longer than 300 mm were discarded. A total of 1,000,000 seeds were placed.

#### Graph theory analysis

GRETNA software^1^ was used to analyse the topological properties of the brain network (31). The brain and cerebellum of the subjects were divided into 116 brain regions using automated anatomical labeling (AAL) atlas. The 116 brain regions of the AAL template are defined as 116 nodes, and the presence of fiber connections between two nodes is defined as the presence of connecting edges. The presence of fiber connections between two nodes was defined as the presence of a connecting edge between the two nodes. The average FA value of all voxels between two connected nodes was calculated and weighted by the corresponding edges to obtain an undirected weighting matrix of FA. A threshold was set for each correlation matrix (Pearson correlation coefficient), which was converted to an undirected binarized matrix with fixed sparsity. The threshold range is selected from 0.1 to 0.4 with a step size of 0.01. The network parameter attributes include global and nodal properties. Global properties are selected as small-world properties and network efficiency. The small-world attributes are the characteristic path length (Lp) is defined as the average shortest path length between all pairs of nodes within a network. This metric serves as a crucial indicator of the network’s overall path efficiency. Specifically, it is evaluated by comparing the characteristic path lengths of the real network against those of 100 randomly generated networks. A shorter Lp indicates enhanced connectivity among nodes, facilitating rapid information transfer, which is critical in medical settings. The clustering coefficient (Cp) quantifies the tendency of nodes in a network to form clusters. It is calculated as the ratio of the clustering coefficient of the real network to that of 100 random networks, providing a measure of the network’s local connectivity. The network efficiency includes: global efficiency (Eglob), which is the inverse of the average of the characteristic path lengths between all pairs of nodes, and is used to measure the global information transfer capability of the network; and local efficiency (Eloc), which is the reciprocal of the shortest average path length in the sub-network consisting of a node and its neighboring nodes, is used to measure the local information transfer capability of the network.

The node characteristics are: nodal efficiency (Ne), which is the reciprocal of the average shortest path length between a node and all other nodes, reflecting the ability of a node to propagate information to other nodes in the network; nodal clustering coefficient (NCp) is a specific measure that quantifies the clustering tendency of an individual node within a network. It reflects how closely connected a node’s neighbors are to each other; betweenness centrality (Bc) refers to the number of shortest paths through a node divided by the number of all possible shortest paths through the node, reflecting the contribution of a node to the shortest paths between all other pairs of nodes; degree centrality (Dc) refers to the most direct metric for portraying the centrality of a node in network analysis. The larger the degree of a node, the higher the degree centrality of this node, which means the more important this node is in the network. Each network parameter was expressed as area under the curve (AUC) in the sparsity range (0.1–0.4), and the computed brain areas with differences were visualized using the BrainNet Viewer^2^ software (32).

#### Adverse events and safety

We strictly followed the trial protocol to judge whether the trial data were outliers or not, rather than making judgments based on our own clinical diagnosis and treatment experience. Researchers should strictly implement the AE (Adverse Events) and SAE (Serious Adverse Event) reporting system and SOPs to enhance the reporting and handling of AEs and SAEs. In this project, electroacupuncture treatment and nuclear magnetic examination had been screened for contraindications. Therefore, no SAE occurred. During electroacupuncture treatment, five subjects reported feeling soreness at the needle site, which was assessed by the acupuncturist to be a normal treatment response, and the soreness disappeared when the resultant electroacupuncture treatment was 2 h old. Four subjects were unable to complete the entire sequence of MRI due to MRI noise, and three subjects were unable to remain still during the examination and the test was discontinued.

### Statistical analysis

All statistical analyses were performed using SPSS version 27.0 (Chicago, IL). The Kolmogorov–Smirnov test was used to assess the normality of each variable. For normally distributed variables, baseline characteristics were presented as mean and standard deviation, while for non-normally distributed variables, baseline characteristics were described using median and interquartile range. Chi-square (*χ*^2^) tests were used to compare gender, education level, lesion location, infarct hemisphere, stroke risk factors, pathological type and acute phase stroke treatments between groups. Analysis of covariance (ANCOVA) was conducted to examine between-group differences, with age, gender, and education level included as covariates. Independent samples t-tests were used to evaluate between-group differences in cognitive test scores and brain network topological properties for normally distributed data. For non-normally distributed data, the Mann–Whitney U test was applied. Pearson correlation analysis was conducted to explore the relationship between cognitive scores and brain network topological properties in PSCI patients, controlling for age, gender, and years of education. A two-tailed *p*-value <0.05 was considered statistically significant. The false discovery rate (FDR) method was applied to correct for multiple comparisons.

## Results

**Figure 2:**
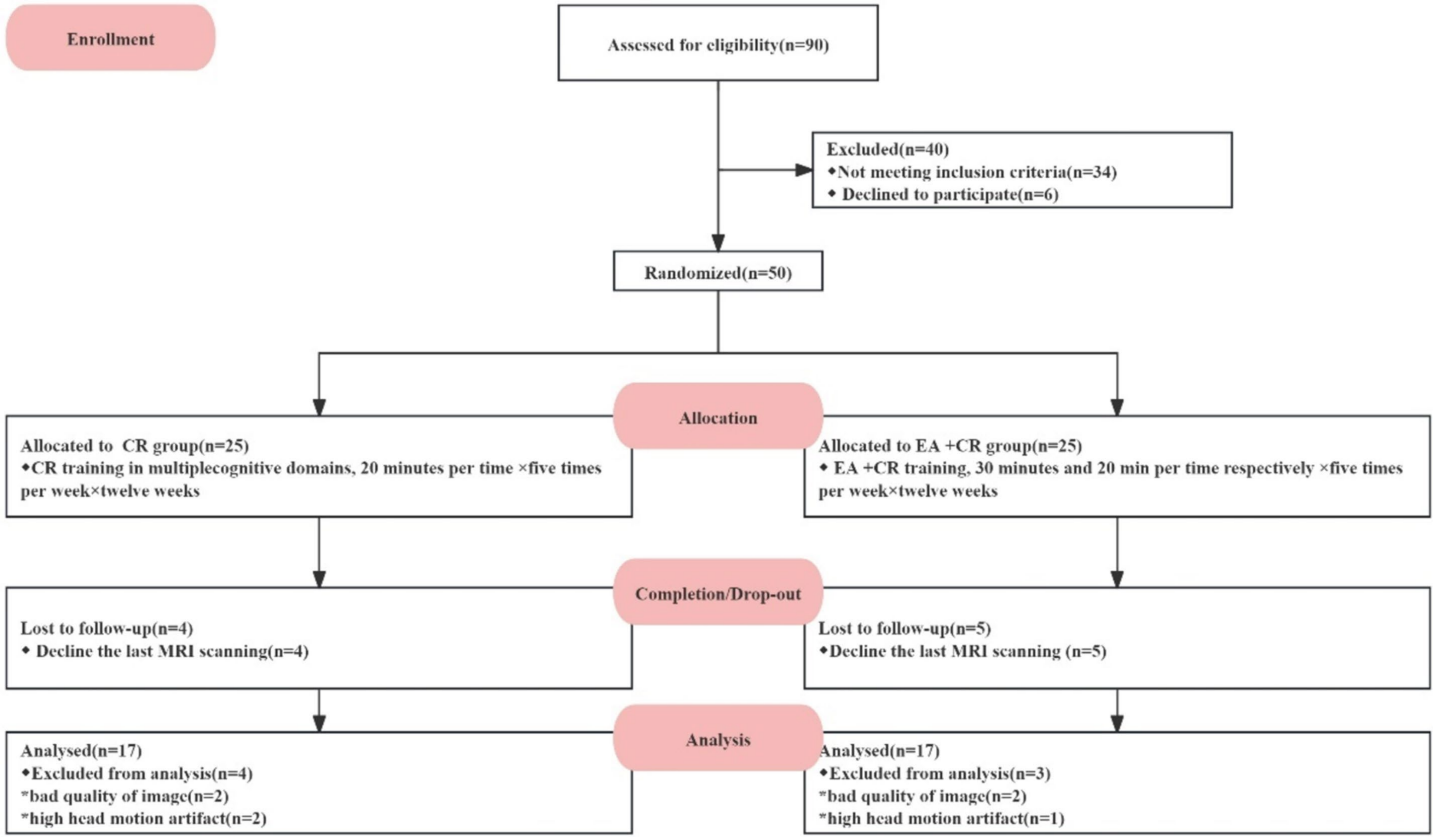
Flow diagram of the trial.

Fifty PSCI patients were screened, and 25 patients were assigned to each group (Cognitive rehabilitation group; EA + cognitive group). Nine patients dropped out because they did not complete a secondary post-treatment MRI scan. Forty-one participants (Cognitive rehabilitation group: *n* = 20; EA + cognitive group: *n* = 21) completed the intervention and clinical assessments and brain scans in both groups. An additional seven patients were excluded from our final analysis due to poor MRI data quality or presence of head motion artifacts -four participants based on DTI data and three based on resting-state image data. No adverse reactions occurred during this study. Ultimately, 34 participants were included in the outcome analysis (see Figure 2).

### Demographic and clinical characteristics of the participants at baseline

There were no significant differences in demographic, clinical characteristics stroke risk factors, stroke type and acute phase stroke treatments between the CR group and the EA + CR group (*p* > 0.05), as shown in Table 1.

**Table 1:** Demographic data of two groups.

Tests		CR group (*n* = 17)	EA + CR group (*n* = 17)	*t*/*χ*^2^	*p*- value
Demographic	Sex (male/female)	13/4	12/5	0.151^a^	0.697
	Age (years)	54.7059 ± 9.3392	49 ± 13.43503	−1.438	0.16
Clinical characteristics	Education (≥12 years/<12 years)	12/5	11/6	0.134^a^	0.714
	Lesion location (C/Sub-C/Both)	4/11/2	1/12/4	2.510^a^	0.285
	Hemisphere of infarction (L/R/Both)	11/5/1	11/6/0	1.091^a^	0.58
Stroke risk factors	Arterial hypertension (yes/no)	1/16	4/13	2.110^a^	0.146
	Diabetes mellitus (yes/no)	11/6	15/2	2.615^a^	0.106
	Dyslipidaemia (yes/no)	14/3	15/2	0.654^a^	0.419
	Smoking (yes/no)	10/7	10/7	0.000^a^	1
	Obesity(yes/no)	16/1	15/2	0.366^a^	0.545
	Drink (alcohol) (yes/no)	12/5	12/5	0.000^a^	1
Pathological type and acute phase stroke treatments	Stroke type (IS/HS)	5/12	7/10	0.515^a^	0.473
	Intravenous thrombolytic treatment (yes/no)	13/4	16/1	2.110^a^	0.146

Plus–minus are mean ± standard deviation. The comparisons of ages between the two groups were performed using independent sample t test. The p value for demographics in the two groups was obtained using a Chi-square test. *p* < 0.05 was considered significant. ^a^*χ*^2^ value. EA, Electropuncture; C, Cortical; Sub-C, Subcortical; L, Left hemisphere; R, Right hemisphere; IS, Ischemic stroke; HS, Hemorrhagic stroke; CR, cognitive rehabilitation group; EA, electroacupuncture.

### Analysis of the results of the cognitive assessment in the two groups

**Figure 3:**
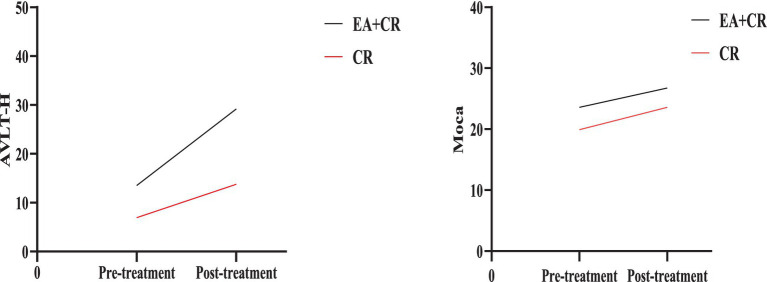
Comparison of AVLT-H and Moca between EA + CR group and CR group pre- and post-treatment. EA, electroacupuncture; CR, cognitive rehabilitation.

The study results indicate that the EA + CR group demonstrated significantly greater improvements than the CR group in several cognitive domains, with corrections for FDR. Specifically, the EA + CR group showed superior performance in the MoCA total score (*p* < 0.001) and AVLT-H total score (*p* = 0.004; Figure 3). Significant differences were also observed in naming (*p* = 0.022), attention (*p* < 0.001), delayed recall (MoCA sub-item delayed recall, *p* < 0.001, the AVLT-H sub-items 5 min short delayed recall, *p* < 0.001 and 15 min long delayed recall, *p* < 0.001) and orientation (*p* = 0.018), as summarized in Table 2.

**Table 2:** Comparison of cognitive results between the two groups.

Variables	CR group	EA + CR group	*t* value	*p* value	FDR
Moca	3.6429 ± 2.06089	11.4667 ± 5.56605	−4.947	<0.001	<0.001
Executive	0.3571 ± 0.49725	0.3571 ± 0.49725	0.13	0.897	0.897
Visuospatial	0.5714 ± 0.85163	0.3333 ± 0.48795	−1.669	0.107	0.191
Naming	0.0714 ± 0.26726	1.3333 ± 1.49603	−2.947	0.007	0.022
Attention	0.5714 ± 0.64621	0.7333 ± 0.79881	−4.449	<0.001	<0.001
Language	0.5 ± 0.65044	2.4 ± 1.40408	−2.313	0.029	0.066
Abstraction	0.2143 ± 0.42582	1.2 ± 0.94112	−2.497	0.019	0.053
Delayed recall	0.6429 ± 0.63332	0.8 ± 0.7746	−5.292	<0.001	<0.001
Orientation	0.7143 ± 0.82542	2.5333 ± 1.18723	−3.04	0.005	0.018
AVLT-H	6.8235 ± 2.6513	15.6471 ± 9.66269	−3.631	0.001	0.004
Immediate memory1	1.5882 ± 1.00367	3.1176 ± 2.4208	−2.406	0.022	0.055
Immediate memory 2	2.0588 ± 1.02899	2.8824 ± 1.93269	−1.551	0.131	0.218
Immediate memory 3	1.8235 ± 0.80896	2.5294 ± 1.80685	−1.47	0.151	0.236
Short delayed recall of 5 min	0.7647 ± 0.75245	3.5294 ± 2.64853	−4.14	<0.001	<0.001
Long delayed recall of 55 min	0.5882 ± 0.61835	3.5882 ± 2.57534	−4.67	<0.001	<0.001
Digit span test(DST)	1.8824 ± 1.45269	2 ± 2.06155	−0.192	0.849	0.897
Forward	0.9412 ± 0.89935	1 ± 1.11803	−0.169	0.867	0.897
Backward	0.9412 ± 0.82694	1 ± 1.5	−0.142	0.888	0.897
Aphasia Screening Scale	20.5294 ± 9.13864	25.8235 ± 17.88238	−1.087	0.285	0.419
Auditory comprehension	4.9412 ± 3.88057	6.7647 ± 6.60993	−0.981	0.334	0.439
Written language understanding	2.4706 ± 1.32842	4.2353 ± 4.11597	−1.682	0.102	0.191
Sign language understanding	0.4706 ± 0.87447	1.4118 ± 1.97037	−1.8	0.081	0.169
Oral expression	5.9412 ± 3.00979	6.5882 ± 5.42055	−0.43	0.67	0.798
Written expression	5.4118 ± 3.44708	6 ± 3.53553	−0.491	0.627	0.784
Sign language expression	1.2941 ± 1.57181	0.8235 ± 1.0146	1.037	0.307	0.426

Values are mean ± standard deviation. The comparisons of cognition between the two groups were performed using *t*-tests. Because some of these subjects were unable to assess MoCA due to their inability to speak, the number of MoCA score counters differed, *n* = 14 in the CR group, *n* = 15 in the EA + CR group, and *n* = 17 in each of the two groups for the rest of the scale counters. AVLT-H: Auditory verbal learning test Hua Shan Version; FDR: false discovery rate. *p* < 0.05 was considered significant.

### Analysis of global topological properties

**Figure 4:**
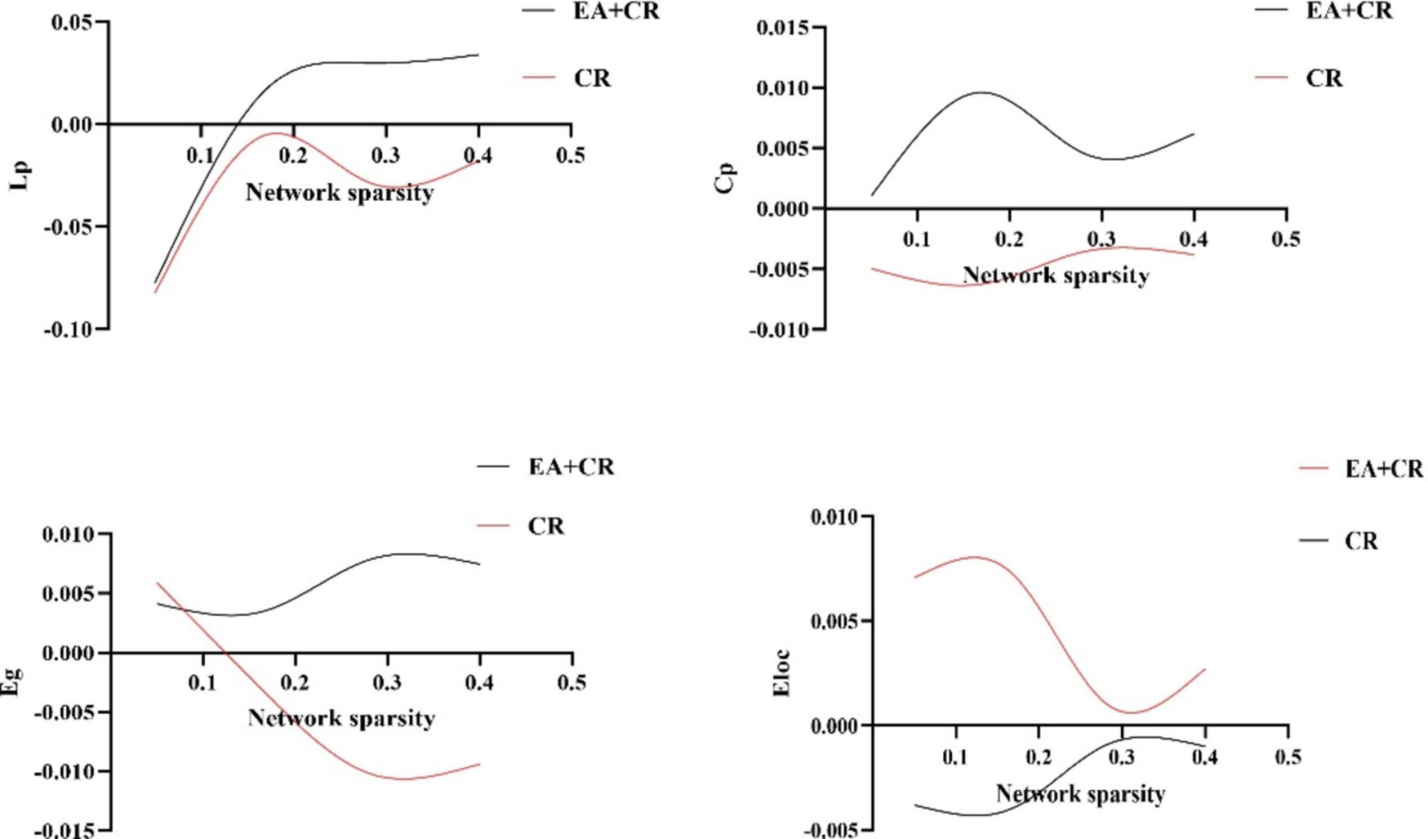
Comparison of global topological properties between EA + CR group and CR group under different network sparsity thresholds. EA, electroacupuncture; CR, cognitive rehabilitation.

**Figure 5:**
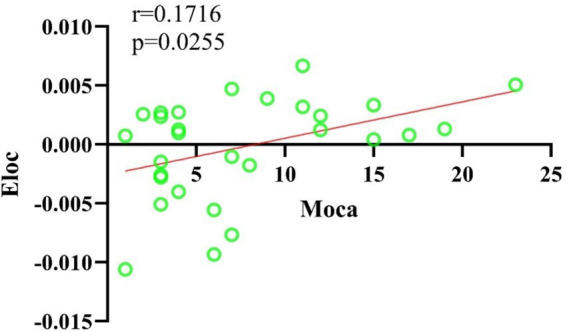
Correlation analysis of Eloc and MoCA scores in the EA + CR group.

In this study, the Cp and Eloc values for the EA + CR group were significantly higher than those for the CR group (*p* < 0.05), suggesting that the combined intervention improved local brain information processing. Lp was also significantly higher in the EA + CR (*p* < 0.05), while no significant difference in Eglob was observed between the two groups (*p* > 0.05; see Table 3; Figure 4). Correlation analyses were done between each of the four indicators and MoCA scores in the EA + CR group, and it was found that Eloc was positively and statistically significantly correlated with MoCA (*r* = 0.1716, *p* = 0.0255; Figure 5), while the rest of the indicators were not statistically significant (Supplementary Table S1). No statistically significant correlation was found between AVLT-H and global attribute correlation (Supplementary Table S2). This indicates that while the intervention enhanced local information processing, it had minimal impact on global information integration.

**Table 3:** Comparison of global topological properties between CR group and EA + CR group.

Variables	CR group (*n* = 17)	EA + CR group (*n* = 17)	*t* value	*p* value	FDR
Lp	−0.0019 ± 0.00332	0.0020 ± 0.00569	2.426	0.003	0.012
Cp	−0.0016 ± 0.00572	0.0021 ± 0.00348	2.331	0.026	0.035
Eglob	0.0015 ± 0.00317	−0.0008 ± 0.00534	−1.569	0.129	0.129
Eloc	−0.0019 ± 0.00332	0.002 ± 0.00569	2.426	0.023	0.035

Values are mean ± standard deviation. The comparisons of global topological properties between the two groups were performed using *t*-tests. Cp, clustering coefficient; Lp, characteristic path length; Eglob, global efficiency; Eloc, local efficiency; *p* value of < 0.05 was considered statistically significant. EA, electroacupuncture; CR, cognitive rehabilitation; FDR, false discovery rate.

### Group differences in nodal efficiency

**Figure 6:**
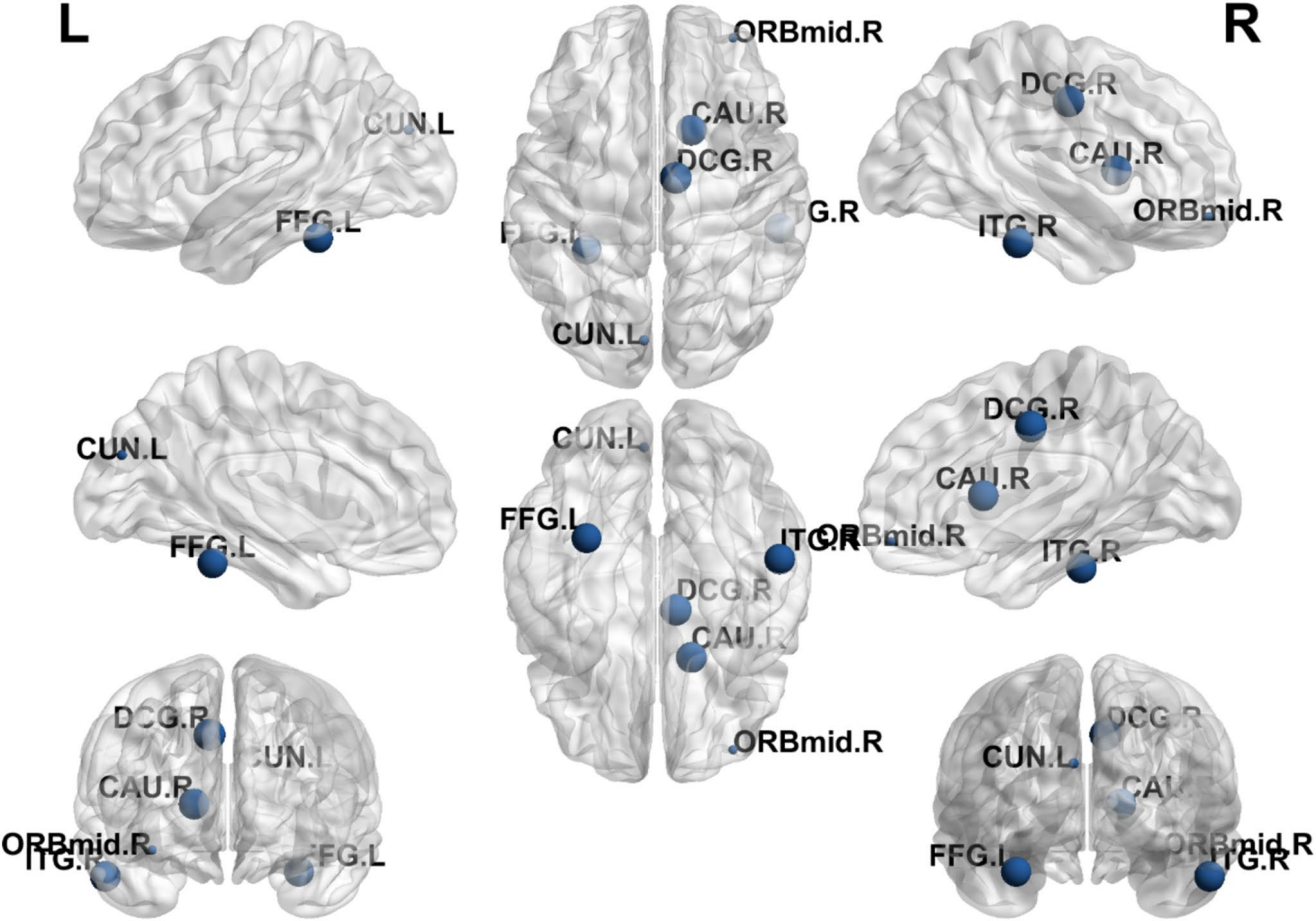
Visualization of nodal efficiency comparing the EA + CR group with the CR group. The blue spherical markers indicate the brain regions with different node clustering coefficients between the EA + CR group and the CR group, and the larger the blue ball is, the more obvious the difference is. The blue spherical markers: brain regions; Size of the blue sphere: Variation range of node clustering coefficients between the two groups; L, Left hemisphere; R, Right hemisphere; ORBmid, Middle frontal gyrus, orbital part; DCG, The medial and Median cingulate; CUN, Cuneus; FFG, fusiform gyrus; CAU.R, Caudatenucleus; ITG.R, inferotemporal gyrus; EA, electroacupuncture; CR, cognitive rehabilitation.

The results showed that the brain regions with significantly higher nodal efficiency in the EA + CR group compared to the CR group were DCG.L, FFG.R, CAU.R and ITG.R. The regions with higher nodal efficiency were mainly located in the frontal lobe, temporal lobe and corpus callosum (Table 4; Figure 6).

**Table 4:** Comparison of nodal efficiency between EA + CR group and CR group.

Region	CR group (*n* = 17)	EA + CR group (*n* = 17)	*t* value	*p* value	FDR
ORBmid.R	3.6718 ± 5.50699	−3.8043 ± 10.68666	−2.564	0.015	0.038
DCG.L	−3.4129 ± 12.41488	8.5326 ± 15.30404	2.499	0.018	0.038
CUN.L	2.1053 ± 9.61331	−7.1164 ± 13.16492	−2.332	0.026	0.038
FFG.R	0.1997 ± 11.70146	12.0077 ± 20.07256	2.095	0.044	0.044
CAU.R	−5.9667 ± 24.76075	14.5658 ± 27.65334	2.281	0.029	0.038
ITG.R	−4.3012 ± 10.93285	4.3732 ± 11.58948	2.245	0.032	0.038

Values are mean ± standard deviation. The comparisons of nodal efficiency between the two groups were performed using *t*-tests. L, Left hemisphere; R, Right hemisphere; ORBmid, Middle frontal gyrus, orbital part; DCG, The medial and Median cingulate; CUN, Cuneus; FFG, fusiform gyrus; CAU.R, Caudatenucleus; ITG.R, inferotemporal gyrus; EA, electroacupuncture; CR, cognitive rehabilitation; FDR, false discovery rate.

### Group differences differences in nodal clustering coefficient

**Figure 7:**
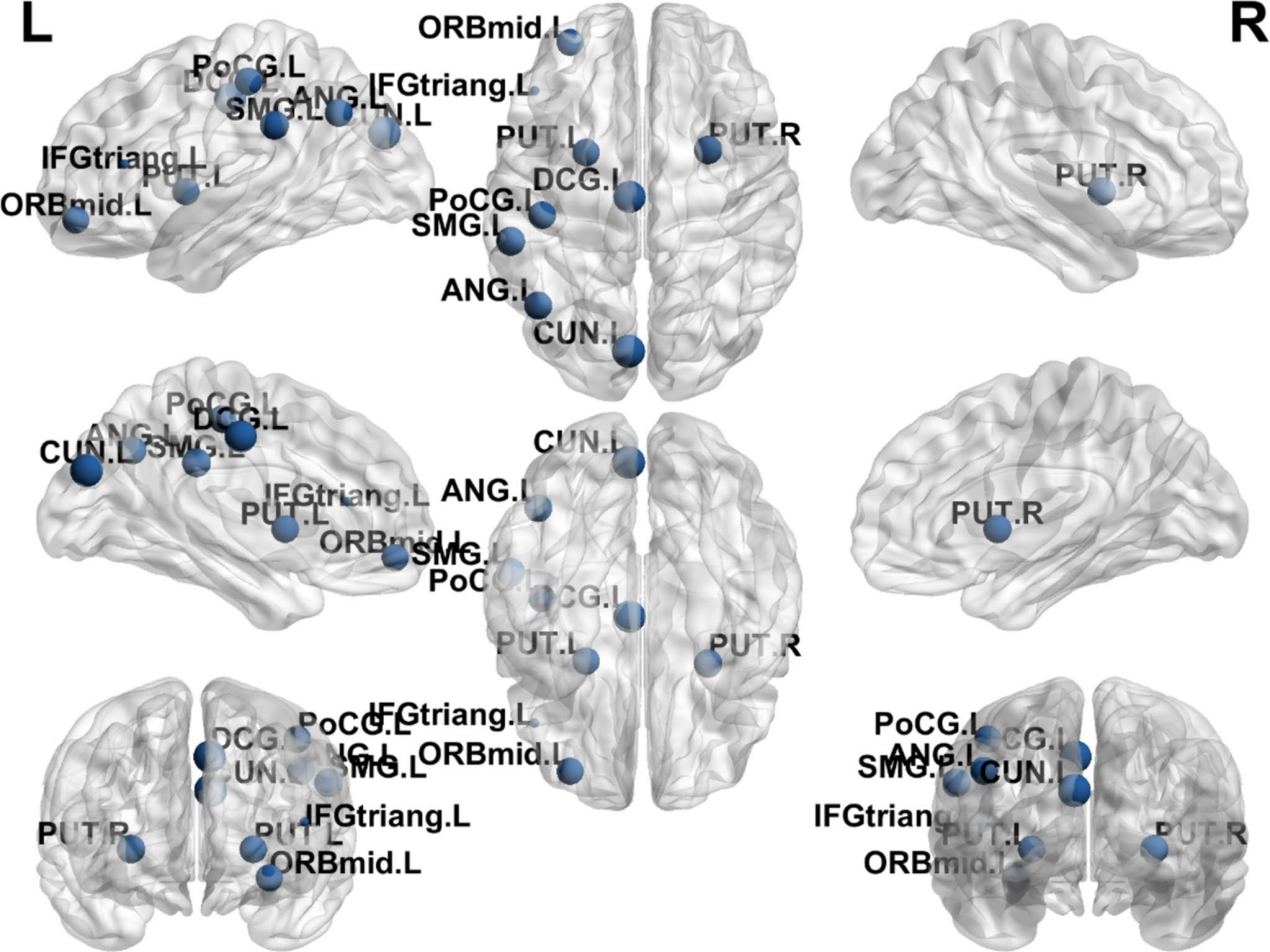
Visualization of node clustering coefficients comparing the EA + CR group with the CR group. The blue ball indicates the brain regions with different node clustering coefficients between the EA + CR group and the CR group, and the larger the blue ball is, the more obvious the difference is. The blue spherical markers: brain regions; Size of the blue sphere: Variation range of node clustering coefficients between the two groups; L, Left hemisphere; R, Right hemisphere; ORBmid, Middle frontal gyrus, orbital part; IFGtriang, Inferior frontal gyrus, triangular part; DCG, The medial and Median cingulate; CUN, Cuneus; PoCG, Postcentral gyrus; SMG, Supramarginal gyrus; ANG, Angular gyrus; PUT, Lenticular nucleus, putamen.

The results showed that the brain regions with significantly higher nodal clustering coefficient in the EA + CR group compared to the CR group were ORBmid.L, DCG.R, CUN.R, PoCG.R, SMG.L, ANG.L, PUT.L and PUT.R. The regions with increased Ncp were mainly located in the prefrontal lobe, parietal lobe, occipital lobe and corpus callosum (Table 5; Figure 7).

**Table 5:** Comparison of nodal clustering coefficient between EA + CR group and CR group.

Region	CR group (*n* = 17)	EA + CR group (*n* = 17)	*t* value	*p* value	FDR
ORBmid.L	−0.0152 ± 0.04594	0.0131 ± 0.03275	2.066	0.047	0.047
IFGtriang.L	0.0323 ± 0.03662	0.0015 ± 0.03634	−2.456	0.02	0.040
DCG.R	−0.0086 ± 0.02399	0.0168 ± 0.01986	3.36	0.002	0.009
CUN.R	−0.024 ± 0.03203	0.0231 ± 0.04367	3.589	0.001	0.009
PoCG.R	−0.0183 ± 0.02206	0.002 ± 0.0306	2.218	0.034	0.043
SMG.L	−0.0229 ± 0.04279	0.0155 ± 0.04581	2.527	0.017	0.040
ANG.L	−0.0019 ± 0.02835	0.0178 ± 0.02175	2.279	0.029	0.043
PUT.L	−0.0026 ± 0.01586	0.0156 ± 0.03075	2.17	0.038	0.043
PUT.R	−0.0063 ± 0.01808	0.0113 ± 0.02416	2.412	0.022	0.040

Values are mean ± standard deviation. The comparisons of nodal clustering coefficient between the two groups were performed using *t*-tests. L, Left hemisphere; R, Right hemisphere; ORBmid, Middle frontal gyrus, orbital part; IFGtriang, Inferior frontal gyrus, triangular part; DCG, The medial and Median cingulate; CUN, Cuneus; PoCG, Postcentral gyrus; SMG, Supramarginal gyrus; ANG, Angular gyrus; PUT, Lenticular nucleus, putamen; FDR, false discovery rate.

### Group differences differences in betweenness centrality

**Figure 8:**
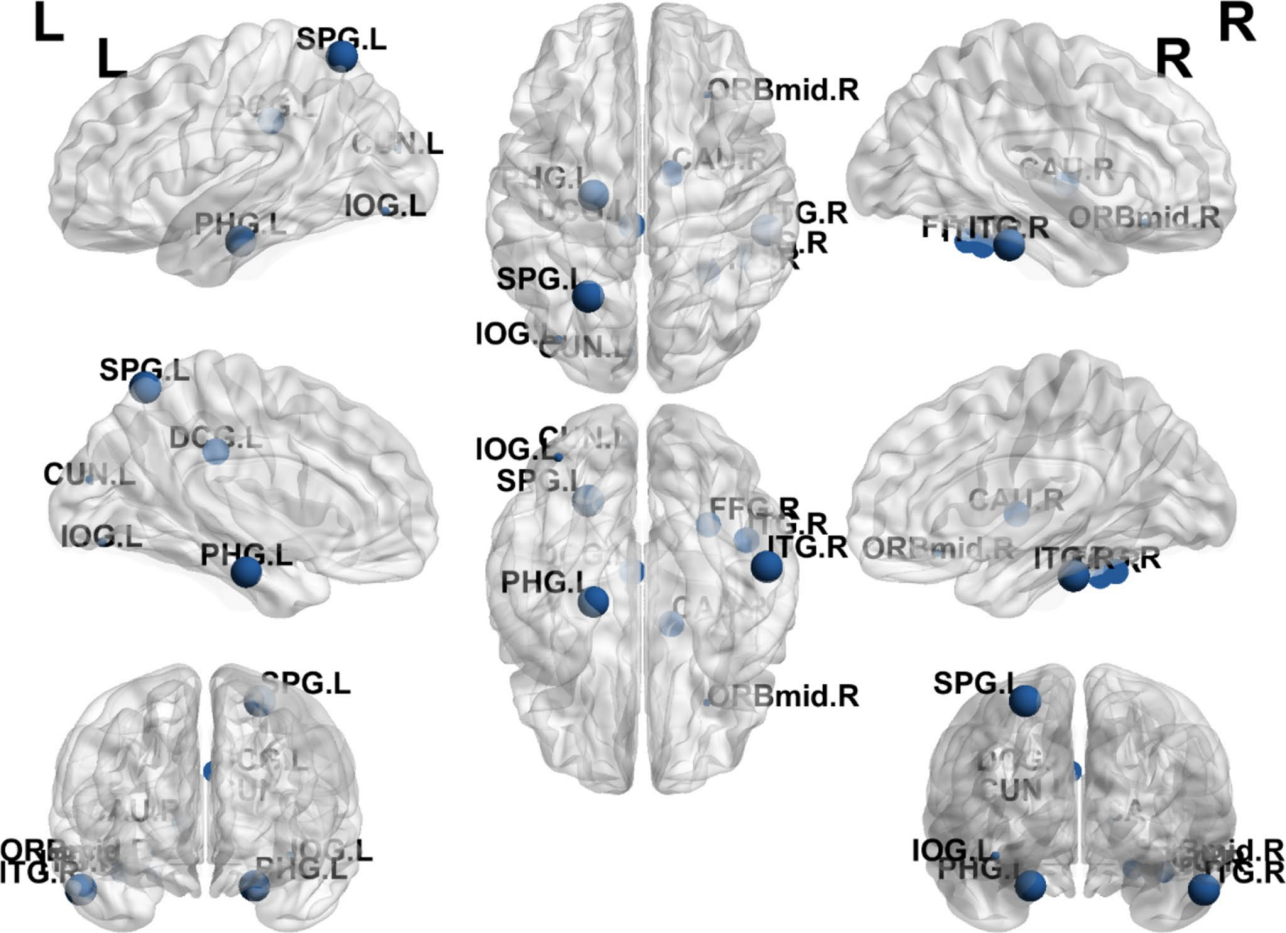
Visualization of Bc comparing the EA + CR group with the CR group. The blue ball indicates the brain regions with different node clustering coefficients between the EA + CR group and the CR group, and the larger the blue ball is, the more obvious the difference is. The blue spherical markers: brain regions; Size of the blue sphere: Variation range of node clustering coefficients between the two groups; L, Left hemisphere; R, Right hemisphere; PHG, Parahippocampal gyrus; IOG, Inferior occipital gyrus; SPG, Superior parietal gyrus; ITG, Inferior temporal gyrus; FDR, false discovery rate.

The results showed that the brain regions with significantly higher Bc in the EA + CR group compared to the CR group were PHG.L, SPG.L and ITG.R. The areas with increased Bc were mainly located in the parietal lobe and temporal lobe (Table 6; Figure 8).

**Table 6:** Comparison of betweenness centrality between EA + CR group and CR group.

Region	CR group (*n* = 17)	EA + CR group (*n* = 17)	*t* value	*p* value	FDR
PHG.L	10.1801 ± 8.14259	22.0602 ± 22.3828	2.057	0.048	0.049
IOG.L	7.6564 ± 3.85271	4.463 ± 3.74224	−2.451	0.02	0.049
SPG.L	37.369 ± 32.07077	64.1867 ± 37.77299	2.231	0.033	0.049
ITG.R	9.6347 ± 7.53939	18.1128 ± 13.95337	2.204	0.035	0.049

Values are mean ± standard deviation. The comparisons of Bc between the two groups were performed using *t*-tests. L, Left hemisphere; R, Right hemisphere; PHG, Parahippocampal gyrus; IOG, Inferior occipital gyrus; SPG, Superior parietal gyrus; ITG, Inferior temporal gyrus; FDR, false discovery rate.

### Group differences differences in degree centrality

**Figure 9:**
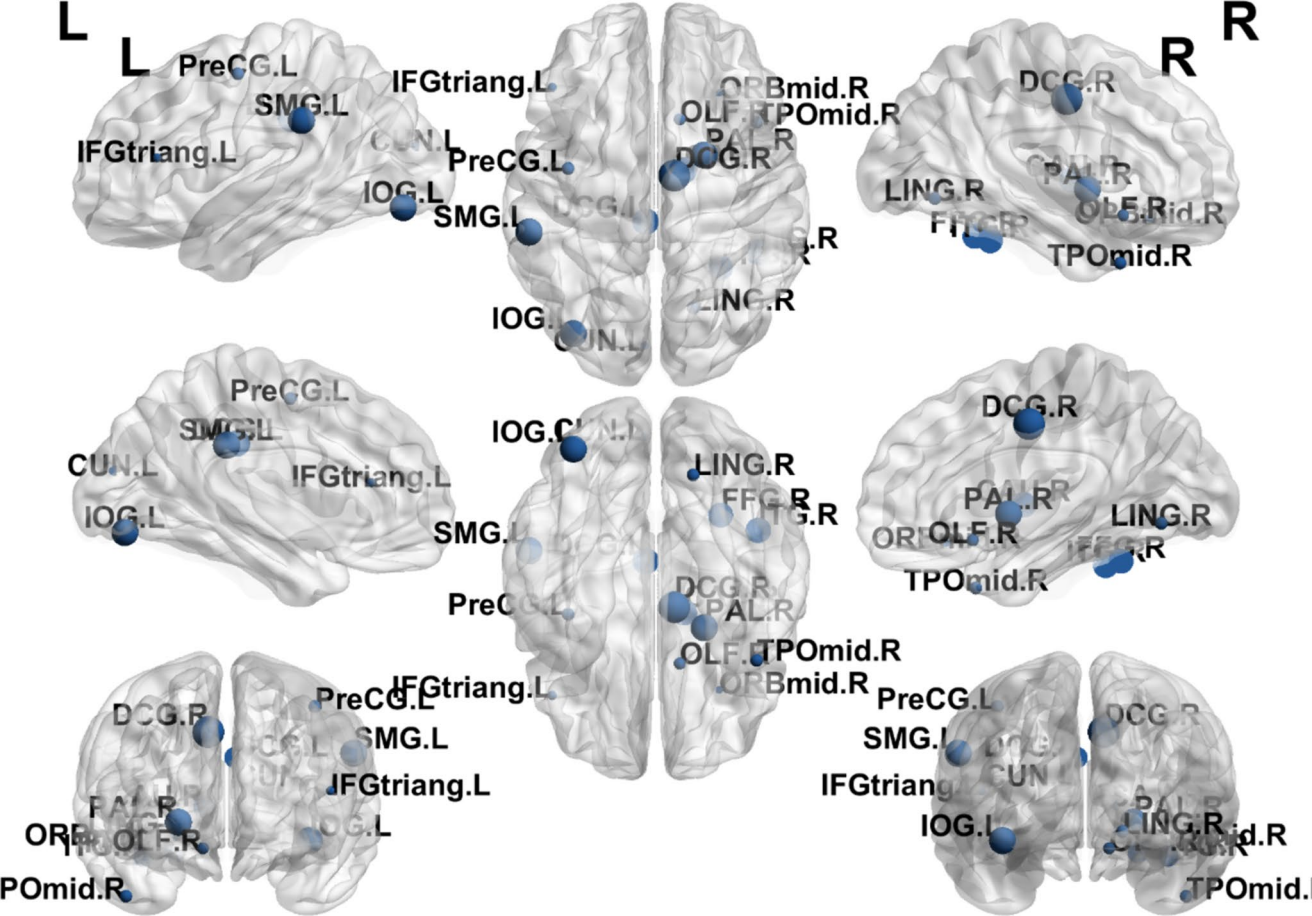
Visualization of Dc comparing the EA + CR group with the CR group. The blue ball indicates the brain regions with different node clustering coefficients between the EA + CR group and the CR group, and the larger the blue ball is, the more obvious the difference is. The blue spherical markers: brain regions; Size of the blue sphere: Variation range of node clustering coefficients between the two groups; L, Left hemisphere; R, Right hemisphere; PreCG, Precental gyrus; IFGtriang, Inferior frontal gyrus, triangular part; OLF, Olfactory cortex; DCG, Median cingulate and paracingulate gyri; LING, Lingual gyrus; IOG, Inferior occipital gyrus; SMG, Supramarginal gyrus; PAL, Lenticular nucleus, pallidum; TPOmid, Temporal pole: middle temporal gyrus; FDR, false discovery rate.

The results showed that the brain regions with significantly higher nodal efficiency in the electroacupuncture + cognitive rehabilitation group compared to the cognitive rehabilitation group were Median cingulate and paracingulate gyri R, Inferior occipital gyrus L, Supramarginal gyrus L and Lenticular nucleus R. The regions with increased degree centrality were mainly located in the frontal lobe, parietal lobe, occipital lobe and corpus callosum (Table 7; Figure 9).

**Table 7:** Comparison of degree centrality between EA + CR group and CR group.

Region	CR group (*n* = 17)	EA + CR group (*n* = 17)	*t* value	*p* value	FDR
PreCG.L	0.0276 ± 0.15659	−0.1434 ± 0.29824	−2.093	0.044	0.049
IFGtriang.L	0.249 ± 0.51388	−0.2137 ± 0.32883	−3.127	0.004	0.026
OLF.R	0.0802 ± 0.47826	−0.2498 ± 0.37138	−2.247	0.032	0.049
DCG.R	−0.177 ± 0.30521	0.1952 ± 0.30528	3.555	0.001	0.013
LING.R	0.0747 ± 0.22161	−0.0815 ± 0.22343	−2.047	0.049	0.049
IOG.L	−0.2162 ± 0.35583	0.0409 ± 0.32754	2.192	0.036	0.049
SMG.L	−0.17 ± 0.43343	0.2955 ± 0.74134	2.235	0.032	0.049
PAL.R	−0.0766 ± 0.23419	0.0936 ± 0.22395	2.166	0.038	0.049
TPOmid.R	0.1155 ± 0.34895	−0.1077 ± 0.2708	−2.084	0.045	0.049

Values are mean ± standard deviation. The comparisons of Bc between the two groups were performed using *t*-tests. L, Left hemisphere; R, Right hemisphere; PreCG, Precental gyrus; IFGtriang: Inferior frontal gyrus, triangular part; OLF, Olfactory cortex; DCG, Median cingulate and paracingulate gyri; LING, Lingual gyrus; IOG, Inferior occipital gyrus; SMG, Supramarginal gyrus; PAL, Lenticular nucleus, pallidum; TPOmid, Temporal pole, middle temporal gyrus; FDR, false discovery rate.

## Discussion

### Improvement of cognitive function and brain network topology through electroacupuncture in PSCI

There have been many previous studies exploring the correlation between brain structural remodeling and brain function. Healthy individuals, those with progressive moderate cognitive impairment and those with stable moderate cognitive impairment exhibited different brain network topological properties characterized by significant differences in intra- and inter-module connectivity between the three groups and correlated with their cognitive function (9). Altered network topological properties in the left supplementary motor area of the brain and the frontoparietal lobe may be a potential pathological mechanism for depression. Impaired information integration of neural networks, especially the disassociation of the frontoparietal network, may be related to the recurrence of depression (33). In this study, we observed that patients in the EA combined with CR group demonstrated significant improvements in several cognitive domains compared to those receiving cognitive rehabilitation alone. These improvements were noted across the total Montreal Cognitive Assessment (MoCA) score, as well as specific domains such as situational memory (AVLT-H), naming, attention, memory, and orientation. Then, we explored the mechanism of electroacupuncture action using graph theoretic methods and found that there was a difference in the change of topological attributes of brain network in the EA + CR training group than in the CR training group, which was manifested by the increase of both global attributes (Cp and Eloc), and the change of Eloc in EA + CR group was significantly and positively correlated with the change of MoCA, which indicated that electroacupuncture mainly affects the local information processing ability of the brain, and may be the promotion of the improvement of the cognitive function of Neural mechanism.

Additionally, we observed increased nodal efficiency (Ne) in regions predominantly located in the frontal and temporal lobes. Furthermore, increases in nodal clustering coefficient (Ncp) were found in the prefrontal, parietal, and occipital lobes, while betweenness centrality (Bc) were raised in the parietal and temporal lobes and degree centrality (Dc) were elevated in the frontal, parietal, and occipital lobes. These regions are closely associated with cognitive functions, implying that electroacupuncture may enhance brain functional connectivity, thereby promoting cognitive recovery in patients with PSCI. These findings offer new insights into the neurobiological mechanisms of electroacupuncture in facilitating recovery from PSCI.

The superiority of electroacupuncture in improving cognitive dysfunction has also been confirmed in previous studies. EA of Baihui (GV 20), Shishencong (EX-HN1), Fengchi (GB 20), and Shenting (GV 24) combined with computer-based cognitive rehabilitation can restore cognitive function in patients with mild cognitive impairment (34). The potential biological mechanisms of electroacupuncture in improving cognition have now been explored. EA stimulation of Shangxing (GV23) and Fengfu (GV16) can improve cognitive impairment in rats by suppressing oxidative stress and neuroinflammation (35); Another study found that EA downregulated LC3 II/LC3 I and autophagosomes, and upregulated the expression of p62, mTOR and Beclin-1 in MCAO rats. It is suggested that electroacupuncture promotes the repair of neuronal cells through the regulation of autophagy network system (36). This study is the first to explore the mechanism of action of electroacupuncture in PSCI in a clinical study.

### Differences in global brain network properties and their mechanisms

In addition to the DTI technique mentioned in this study, other means of studying brain networks in the post-stroke brain have been used in the past. After stroke, the central nervous system usually reorganizes brain networks to restore damaged functions (37). In detecting functional remodeling of the brain, quantitative Electroencephalography (qEEG) is one of the more well-studied and commonly used techniques in clinical practice. A meta-analysis was performed to summarize the use of QEEG in stroke. The results found that the QEEG-derived indices DAR (delta-alpha ratio) and DTABR (delta-theta-alpha-beta ratio) were positively correlated with post-stroke functioning (38). Another study used quantitative EEG to explore the mechanisms by which bilateral robotics promotes recovery after stroke and found that elevated pdBSI (pairwise derived brain symmetry) was accompanied by improved upper limb function after stroke (39). However, qEEG is analyzed primarily by recording electrical activity in the cerebral cortex, so its focus is usually on cortical (cerebral cortex) areas rather than subcortical brain regions (40). The DTI can effectively test the structural connections between the cerebral cortex and subcortical brain regions and is particularly suitable for studying the connections between white matter fiber tracts and brain regions. PSCI includes cognitive deficits caused by both cortical and subcortical impairments. Cortical damage primarily affects higher cognitive functions, such as language, memory, and executive functions, whereas subcortical damage is more involved in emotion regulation, attention, and basic cognitive processes (e.g., basal ganglia, hippocampus, and thalamus) (41). Both impairments may intersect with each other and together lead to cognitive decline after stroke. By tracking and analyzing these connections, DTI can help researchers understand the interactions between cortical and subcortical brain regions in terms of neurological function, cognition and motor control (42).

Cognitive impairment is closely linked to disruptions in brain network structures. The severity of cognitive decline has been associated with greater disorganization of brain functional connectivity networks. The brain exhibits a modular, small-world network architecture, which facilitates efficient information transmission at minimal wiring costs. The characteristic path length (Lp) reflects the optimal distance for information transmission between two nodes, with shorter Lp corresponding to faster information transfer. In contrast, Cp reflects short-range information transmission capacity, where higher C*p* values indicate enhanced local information processing.

A brain network with high Cp and low Lp is said to exhibit small-world properties, ensuring both local and global efficiency in information transmission. This configuration enables the brain to maintain highly interconnected hub regions, which are critical for cognitive function. The global efficiency (Eg) and Eloc further ensure the balance between segregation and integration in information processing. Eg measures the brain’s capacity for global information transfer, while Eloc reflects efficiency at the level of individual nodes. Higher values of Eg and Eloc signify improved global and local network efficiency, respectively.

Patients with more severe cognitive impairment exhibited reductions in Eglob, Eloc, and Cp, accompanied by increases in Lp (43). These changes indicate a decline in information transfer efficiency, reduced network connectivity, and fewer inter-nodal connections. It has been found that PSCI language-related brain regions show an increase in Lp and a decrease in small world (44). Eloc and Cp can measure the efficiency of local information transfer. Compared with the CR group alone, Eloc and Cp increased in the EA + CR group, and Eloc was positively correlated with the change of MoCA. This suggests that electroacupuncture can increase the local information transfer efficiency of brain networks and may be a potential mechanism for cognitive improvement.

Lp was increased in the EA+ CR group, the significance of which needs to be further explored. Son et al. (45) found that Lp was significantly lower in the MCI group compared to the NC group, similarly, Zhang et al. (46) study also showed that Lp was significantly shorter in the MCI group. And according to the significance of Lp, the shorter the Lp, the faster the information propagation. While in pathological conditions, Lp was shortened instead, maybe Lp response to cognitive function is not sensitive, or there are other favorable meanings that we need to continue to explore. Or maybe it is because of errors arising from patient screening conditions or DTI research techniques.

### Differences in nodal brain network properties and their mechanisms

Nodal efficiency (Ne) measures the fault tolerance of a network, indicating the extent to which neighboring nodes can continue exchanging information if a node is removed. As cognitive impairment worsens, larger areas of the brain show reduced Ne. Nodal clustering coefficient (Ncp) measures the degree of grouping in the network; the higher the Ncp, the more likely that the node’s neighbors are in a functional module with each other. Betweenness centrality (Bc) reflects a node’s role as a bridge in the network, influencing the flow of information between other nodes. Increased Bc may again reflect vicarious network reorganization to increase effective information transfer in PSCI patients (44). While degree centrality (Dc) represents the number of connections a node has within the network, indicating its capacity to disseminate informatetwork, indicatingition (47–50).

In our analysis, the EA + CR group exhibited increases in Ne, Ncp, Bc, and Dc across regions involved in cognitive functions, including the frontal, parietal, temporal, and occipital lobes. The frontal lobes, particularly the prefrontal cortex, are critical for higher-order cognitive functions such as decision-making, attention, and problem-solving (51, 52). The median cingulate and paracingulate gyri (DCG) show Ne and Dc enhancement, which is the main node of the default mode network (DMN) and is the most and best studied of the resting state network (RSN) (53). DMN has been a hot area of human cognitive research since its discovery. It is most active when the person is resting instead, such as walking, reminiscing, thinking about the future and other internally centered thought processes. The DMN plays a role in several cognitive functions, including memory, social cognition, semantic comprehension, and emotional processing (54). The ability of DMN regional brain network to exchange information increased in the electroacupuncture intervention group, suggesting that electroacupuncture is valuable for the recovery of self-perception and emotion in post-stroke patients. The parietal lobes, essential for attention, spatial reasoning, and distinguishing self from others, also showed increased nodal properties (55). Specifically, these functions are primarily performed by the prefrontal cortex in the frontal lobe. The second Broca’s area is located in the frontal lobe and is associated with the motor components of speech production. The parietal lobes are crucial in tasks such as attention, spatial reasoning, and distinguishing self from others. The posterior parietal cortex, which integrates sensory information and supports complex cognitive tasks such as language comprehension and motor planning. Dysfunction in this region has been associated with cognitive deficits in PSCI (56). One notable parietal brain gyrus was the left supramarginal gyrus, which had increased Ncp and Dc. The left supramarginal gyrus is one of the key nodes of the short-term memory network, which stores abstract representations of sequential order information independently of content information (i.e., the nature of the item to be remembered) (57). The temporal lobe plays a crucial role in object recognition and works with other brain structures to form both new and long-term memories. It is also responsible for emotional processing, memory retrieval, and language comprehension (58), we noted increases in Ne and Bc within the inferior temporal gyrus. This region contributes to semantic memory, language, and visual processing, and reduced synaptic density here has been linked to cognitive dysfunction (55). Additionally, the occipital lobes, which are responsible for processing visual stimuli and spatial reasoning, exhibited increased nodal centrality, underscoring their role in cognitive recovery (59).

### Study limitations

Several limitations in this study must be addressed. First, the small sample size may have limited the statistical power to detect more subtle differences between groups. Despite including patients with varying levels of cognitive impairment, the results may not generalize widely. Future studies should recruit larger samples to improve statistical robustness. Second, the number and location of brain lesions varied among participants, which may have influenced the results. Future research should aim for greater lesion homogeneity. Third, diffusion tensor imaging (DTI) technology has inherent limitations, such as spatial resolution, which prevent the accurate visualization of smaller fiber bundles or regions where fiber bundles intersect. This limitation may have affected the accuracy of our brain network analyses. Future studies should focus on improving the methods used for constructing brain networks to achieve higher accuracy and sensitivity. Additionally, current research primarily focuses on the topological properties of brain networks, while the formation and dynamic evolution of these networks remain underexplored. Further investigation into these mechanisms is warranted. Fourth, this study focuses on short-term outcomes over 12 weeks. It would be valuable to discuss whether the observed improvements in cognitive function and brain topography are likely to be sustained in the long term. We will address this question in future studies.

## Conclusion

Regarding post-stroke cognitive impairments, we have done previous basic research exploring their pathological mechanisms. We found that post-stroke cognitive impairment is related to the structure and inflammation level of brain white matter, which is the place where nerve fibers gather inside the brain, and the better the recovery of brain white matter and the lower the inflammation level, the better the cognitive function (60). This suggests that the brain white matter has remodeling properties. And the research content of this paper and previous studies can confirm each other that brain white matter remodeling may be associated with the change of brain network topological properties. In this study, we found that electroacupuncture combined with cognitive rehabilitation can achieve better cognitive function improvement than cognitive rehabilitation alone. Secondly, electroacupuncture can repair and optimize the brain network structure which is more conducive to the smooth flow of information in the brain, thus promoting the recovery of cognitive dysfunction after stroke. Our study is the first to reveal the mechanisms of electroacupuncture from the perspective of brain network topological properties (DTI analysis). Our findings contribute to a better understanding of how electroacupuncture improves cognitive function and restructures brain networks and highlight the importance of electroacupuncture and its underlying neurological mechanisms in the clinical practice of PSCI.

## Data Availability

The raw data supporting the conclusions of this article will be made available by the authors, without undue reservation.

## Footnotes

^1^ http://www.nitrc.org/projects/gratna

^2^ https://www.nitrc.org/projects/bnv
