# Supplementary material for: Electroacupuncture combined with cognitive rehabilitation outperforms cognitive rehabilitation alone in treating post-stroke cognitive impairment: a randomized controlled trial
Source: Front Neurol. 2025 Jan 29;16:1507475. doi: 10.3389/fneur.2025.1507475 (PMC11814160; doi:10.3389/fneur.2025.1507475)
Supplement: Supplementary file 1 [file Data_Sheet_1.pdf]

Montreal Cognitive Assessment (MoCA)
Version 8.1

Administration and Scoring Instructions

The Montreal Cognitive Assessment (MoCA) was designed as a rapid screening instrument for mild cognitive dysfunction. It assesses different cognitive domains: attention and concentration, executive functions, memory, language, visuoconstructional skills, conceptual thinking, calculations, and orientation. The MoCA may be administered by anyone who understands and follows the instructions, however, only a health professional with expertise in the cognitive field may interpret the results. Time to administer the MoCA is approximately 10 minutes. The total possible score is 30 points; a score of 26 or above is considered normal.

All instructions may be repeated once.

1. Alternating Trail Making:

Administration: The examiner instructs the subject: *"Please draw a line going from a number to a letter in ascending order. Begin here* [point to (1)] *and draw a line from 1 then to A then to 2 and so on. End here* [point to (E)]."

Scoring: One point is allocated if the subject successfully draws the following pattern: 1- A- 2- B- 3- C- 4- D- 5- E, without drawing any lines that cross. Any error that is not immediately self-corrected (meaning corrected before moving on to the Cube task) earns a score of 0. A point is not allocated if the subject draws a line to connect the end (E) to the beginning (1).

2. Visuoconstructional Skills (Cube):

Administration: The examiner gives the following instructions, pointing to the cube: "*Copy this drawing as accurately as you can.*"

Scoring: One point is allocated for a correctly executed drawing.

- Drawing must be three-dimensional.
- All lines are drawn.
- All lines meet with little or no space.
- No line is added.
- Lines are relatively parallel and their length is similar (rectangular prisms are accepted).
- The cube's orientation in space must be preserved.

A point is not assigned if any of the above criteria is not met.

3. Visuoconstructional Skills (Clock):

Administration: The examiner must ensure that the subject does not look at his/her watch while performing the task and that no clocks are in sight. The examiner indicates the appropriate space and gives the following instructions: *"Draw a clock. Put in all the numbers and set the time to 10 past 11."*

Scoring: One point is allocated for each of the following three criteria:

© Z. Nasreddine MD

• Contour (1 pt.): the clock contour must be drawn (either a circle or a square). Only minor distortions are acceptable (e.g., slight imperfection on closing the circle). If the numbers are arranged in a circular manner but the contour is not drawn the contour is scored as incorrect.
• Numbers (1 pt.): all clock numbers must be present with no additional numbers. Numbers must be in the correct order, upright and placed in the approximate quadrants on the clock face. Roman numerals are acceptable. The numbers must be arranged in a circular manner (even if the contour is a square). All numbers must either be placed inside or outside the clock contour. If the subject places some numbers inside the clock contour and some outside the clock contour, (s)he does not receive a point for Numbers.
• Hands (1 pt.): there must be two hands jointly indicating the correct time. The hour hand must be clearly shorter than the minute hand. Hands must be centered within the clock face with their junction close to the clock center.

4. Naming:

Administration: Beginning on the left, the examiner points to each figure and says: *"Tell me the name of this animal."*

Scoring: One point is given for each of the following responses: (1) lion (2) rhinoceros or rhino (3) camel or dromedary.

5. Memory:

Administration: The examiner reads a list of five words at a rate of one per second, giving the following instructions: *"This is a memory test. I am going to read a list of words that you will have to remember now and later on. Listen carefully. When I am through, tell me as many words as you can remember. It doesn't matter in what order you say them."* The examiner marks a check in the allocated space for each word the subject produces on this first trial. The examiner may not correct the subject if (s)he recalls a deformed word or a word that sounds like the target word. When the subject indicates that (s)he has finished (has recalled all words), or can recall no more words, the examiner reads the list a second time with the following instructions: *"I am going to read the same list for a second time. Try to remember and tell me as many words as you can, including words you said the first time."* The examiner puts a check in the allocated space for each word the subject recalls on the second trial. At the end of the second trial, the examiner informs the subject that (s)he will be asked to recall these words again by saying: *"I will ask you to recall those words again at the end of the test."*

Scoring: No points are given for Trials One and Two.

6. Attention:

Forward Digit Span: Administration: The examiner gives the following instructions: "*I am going to say some numbers and when I am through, repeat them to me exactly as I said them.*" The examiner reads the five number sequence at a rate of one digit per second.

Backward Digit Span: Administration: The examiner gives the following instructions: "*Now I am going to say some more numbers, but when I am through you must repeat*

© Z. Nasreddine MD

them to me in the <u>*backward*</u> *order.*" The examiner reads the three number sequence at a rate of one digit per second. If the subject repeats the sequence in the forward order, the examiner may not ask the subject to repeat the sequence in backward order at this point.

<u>Scoring:</u> One point is allocated for each sequence correctly repeated (N.B.: the correct response for the backward trial is 2-4-7).

<u>Vigilance: Administration:</u> The examiner reads the list of letters at a rate of one per second, after giving the following instructions: "*I am going to read a sequence of letters. Every time I say the letter A, tap your hand once. If I say a different letter, do not tap your hand.*"

<u>Scoring:</u> One point is allocated if there is zero to one error (an error is a tap on a wrong letter or a failure to tap on letter A).

<u>Serial 7s: Administration:</u> The examiner gives the following instructions: *"Now, I will ask you to count by subtracting 7 from 100, and then, keep subtracting 7 from your answer until I tell you to stop."* The subject must perform a mental calculation, therefore, (s)he may not use his/her fingers nor a pencil and paper to execute the task. The examiner may not repeat the subject's answers. If the subject asks what her/his last given answer was or what number (s)he must subtract from his/her answer, the examiner responds by repeating the instructions if not already done so.

<u>Scoring:</u> This item is scored out of 3 points. Give no (0) points for no correct subtractions, 1 point for one correct subtraction, 2 points for two or three correct subtractions, and 3 points if the subject successfully makes four or five correct subtractions. Each subtraction is evaluated independently; that is, if the subject responds with an incorrect number but continues to correctly subtract 7 from it, each correct subtraction is counted. For example, a subject may respond "92 – 85 – 78 – 71 – 64" where the "92" is incorrect, but all subsequent numbers are subtracted correctly. This is one error and the task would be given a score of 3.

7. <u>Sentence repetition:</u>

<u>Administration:</u> The examiner gives the following instructions: *"I am going to read you a sentence. Repeat it after me, exactly as I say it* [pause]: ***I only know that John is the one to help today."*** Following the response, say: *"Now I am going to read you another sentence. Repeat it after me, exactly as I say it* [pause]: ***The cat always hid under the couch when dogs were in the room."***

<u>Scoring</u>: One point is allocated for each sentence correctly repeated. Repetitions must be exact. Be alert for omissions (e.g., omitting "only"), substitutions/additions (e.g., substituting "only" for "always"), grammar errors/altering plurals (e.g. "hides" for "hid"), etc.

8. <u>Verbal fluency:</u>

<u>Administration:</u> The examiner gives the following instructions: *"Now, I want you to tell me as many words as you can think of that begin with the letter F. I will tell you to stop after one minute. Proper nouns, numbers, and different forms of a verb are not permitted. Are you ready?* [Pause] [Time for 60 sec.] *Stop."* If the subject names two consecutive

© Z. Nasreddine MD

words that begin with another letter of the alphabet, the examiner repeats the target letter if the instructions have not yet been repeated.

Scoring: One point is allocated if the subject generates 11 words or more in 60 seconds. The examiner records the subject's responses in the margins or on the back of the test sheet.

9. Abstraction:

Administration: The examiner asks the subject to explain what each pair of words has in common, starting with the example: *"I will give you two words and I would like you to tell me to what category they belong to* [pause]: *an orange and a banana.*" If the subject responds correctly the examiner replies: *''Yes, both items are part of the category Fruits.''* If the subject answers in a concrete manner, the examiner gives one additional prompt: *"Tell me another category* to *which these items belong to."* If the subject does not give the appropriate response *(fruits)*, the examiner says: *"Yes, and they also both belong to the category Fruits."* No additional instructions or clarifications are given. After the practice trial, the examiner says: *"Now, a train and a bicycle."* Following the response, the examiner administers the second trial by saying: *"Now, a ruler and a watch."* A prompt (one for the entire abstraction section) may be given if none was used during the example.

Scoring: Only the last two pairs are scored. One point is given for each pair correctly answered. The following responses are acceptable:

- train-bicycle = means of transportation, means of travelling, you take trips in both
- ruler-watch = measuring instruments, used to measure

The following responses are **not** acceptable:

- train-bicycle = they have wheels
- ruler-watch = they have numbers

10. Delayed recall:

Administration: The examiner gives the following instructions: *"I read some words to you earlier, which I asked you to remember. Tell me as many of those words as you can remember."* The examiner makes a check mark (√) for each of the words correctly recalled spontaneously without any cues, in the allocated space.

Scoring: **One point is allocated for each word recalled freely without any cues.**

Memory index score (MIS):

Administration: Following the delayed free recall trial, the examiner provides a category (semantic) cue for each word the subject was unable to recall. Example: ''*I will give you some hints to see if it helps you remember the words, the first word was a body part*.'' If the subject is unable to recall the word with the category cue, the examiner provides him/her with a multiple choice cue. Example: *"Which of the following words do you think it was, NOSE, FACE, or HAND?"* All non-recalled words are prompted in this manner. The examiner identifies the words the subject was able to recall with the help of a cue (category or multiple-choice) by placing a check mark (√) in the appropriate space. The cues for each word are presented below:

© Z. Nasreddine MD

Target Word	Category Cue	Multiple Choice
FACE	body part	nose, face, hand (shoulder, leg)
VELVET	type of fabric	denim, velvet, cotton (nylon, silk)
CHURCH	type of building	church, school, hospital (library, store)
DAISY	type of flower	rose, daisy, tulip (lily, daffodil)
RED	color	red, blue, green (yellow, purple)

* The words in parentheses are to be used if the subject mentions one or two of the multiple choice responses during the category cuing.

Scoring: To determine the MIS (which is a sub-score), the examiner attributes points according to the type of recall (see table below). The use of cues provides clinical information on the nature of the memory deficits. For memory deficits due to retrieval failures, performance can be improved with a cue. For memory deficits due to encoding failures, performance does not improve with a cue.

**MIS scoring**				**Total**
Number of words recalled spontaneously	…	multiplied by	3	…
Number of words recalled with a category cue	…	multiplied by	2	…
Number of words recalled with a multiple choice cue	…	multiplied by	1	…
	**Total MIS** (add all points)			**---/15**

11. Orientation:

Administration: The examiner gives the following instructions: "*Tell me today's date*." If the subject does not give a complete answer, the examiner prompts accordingly by saying: *"Tell me the [year, month, exact date, and day of the week]."* Then the examiner says: *"Now, tell me the name of this place, and which city it is in."*

Scoring: One point is allocated for each item correctly answered. The date and place (name of hospital, clinic, office) must be exact. No points are allocated if the subject makes an error of one day for the day and date.

TOTAL SCORE: Sum all subscores listed on the right-hand side. Add one point for subject who has 12 years or fewer of formal education, for a possible maximum of 30 points. A final total score of 26 and above is considered normal.

Please refer to the MoCA website at www.mocatest.org for more information on the MoCA.

MONTREAL COGNITIVE ASSESSMENT (MOCA®)
Version 8.1 English

Name:
Education: Date of birth:
Sex: DATE:

VISUOSPATIAL / EXECUTIVE			POINTS

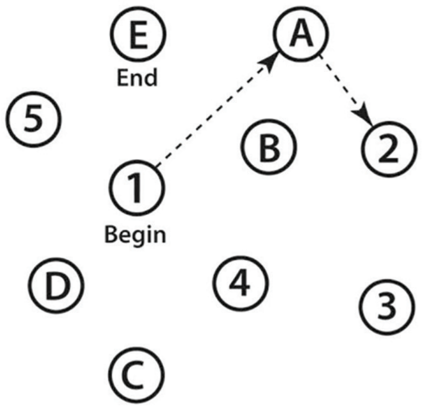

[ ]

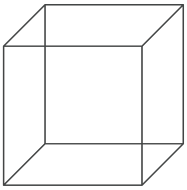

Copy cube

[ ]

Draw CLOCK (Ten past eleven)
(3 points)

[ ] Contour [ ] Numbers [ ] Hands

__/5

NAMING

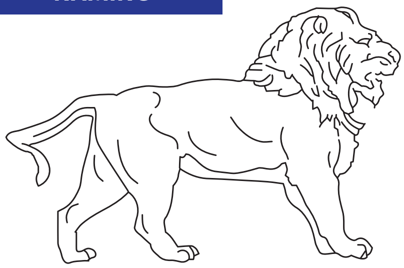

[ ]

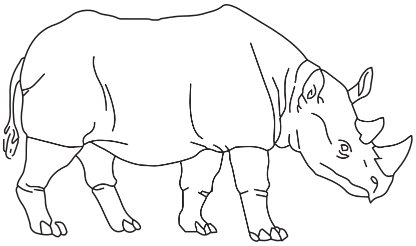

[ ]

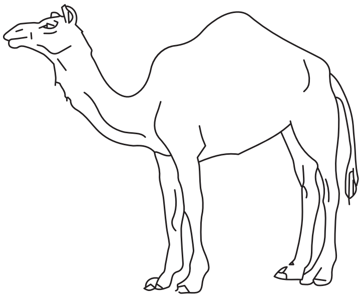

[ ]

__/3

MEMORY

Read list of words, subject must repeat them. Do 2 trials, even if 1st trial is successful. Do a recall after 5 minutes.

	FACE	VELVET	CHURCH	DAISY	RED
1ST TRIAL					
2ND TRIAL					

NO POINTS

ATTENTION

Read list of digits (1 digit/ sec.). Subject has to repeat them in the forward order. [ ] 2 1 8 5 4
Subject has to repeat them in the backward order. [ ] 7 4 2

__/2

Read list of letters. The subject must tap with his hand at each letter A. No points if ≥ 2 errors
[ ] F B A C M N A A J K L B A F A K D E A A A J A M O F A A B

__/1

Serial 7 subtraction starting at 100. [ ] 93 [ ] 86 [ ] 79 [ ] 72 [ ] 65
4 or 5 correct subtractions: **3 pts**, 2 or 3 correct: **2 pts**, 1 correct: **1 pt**, 0 correct: **0**

__/3

LANGUAGE

Repeat: I only know that John is the one to help today. [ ]
The cat always hid under the couch when dogs were in the room. [ ]

__/2

Fluency: Name maximum number of words in one minute that begin with the letter F. [ ] ______ (N ≥ 11 words)

__/1

ABSTRACTION

Similarity between e.g. banana - orange = fruit [ ] train - bicycle [ ] watch - ruler

__/2

DELAYED RECALL

(MIS)	Has to recall words WITH NO CUE	FACE	VELVET	CHURCH	DAISY	RED	Points for UNCUED recall only
X3		[ ]	[ ]	[ ]	[ ]	[ ]	
X2	Category cue						MIS = ____/15
X1	Multiple choice cue						

Memory Index Score (MIS)

__/5

ORIENTATION

[ ] Date [ ] Month [ ] Year [ ] Day [ ] Place [ ] City

__/6

© Z. Nasreddine MD

www.mocatest.org

MIS: /15
(Normal ≥ 26/30)
Add 1 point if ≤ 12 yr edu

TOTAL __/30

Administered by: ______________________________

Training and Certification are required to ensure accuracy

Montreal Cognitive Assessment (MoCA)
Version 8.2

Administration and Scoring Instructions

The Montreal Cognitive Assessment (MoCA) was designed as a rapid screening instrument for mild cognitive dysfunction. It assesses different cognitive domains: attention and concentration, executive functions, memory, language, visuoconstructional skills, conceptual thinking, calculations, and orientation. The MoCA may be administered by anyone who understands and follows the instructions, however, only a health professional with expertise in the cognitive field may interpret the results. Time to administer the MoCA is approximately 10 minutes. The total possible score is 30 points; a score of 26 or above is considered normal.

All instructions may be repeated once.

1. Alternating Trail Making:

Administration: The examiner instructs the subject: *"Please draw a line going from a number to a letter in ascending order. Begin here* [point to (1)] *and draw a line from 1 then to A then to 2 and so on. End here* [point to (E)]."

Scoring: One point is allocated if the subject successfully draws the following pattern: 1- A- 2- B- 3- C- 4- D- 5- E, without drawing any lines that cross. Any error that is not immediately self-corrected (meaning corrected before moving on to the Chair task) earns a score of 0. A point is not allocated if the subject draws a line to connect the end (E) to the beginning (1).

2. Visuoconstructional Skills (Chair):

Administration: The examiner gives the following instructions, pointing to the chair: "*Copy this drawing as accurately as you can.*"

Scoring: One point is allocated for a correctly executed drawing.
- Drawing must be three-dimensional.
- All lines are drawn.
- All lines meet with little or no space.
- No line is added.
- Lines are relatively parallel and their length is similar.
- The chair's orientation in space must be preserved.

A point is not assigned if any of the above criteria is not met.

3. Visuoconstructional Skills (Clock):

Administration: The examiner must ensure that the subject does not look at his/her watch while performing the task and that no clocks are in sight. The examiner indicates the appropriate space and gives the following instructions: *"Draw a clock. Put in all the numbers and set the time to 10 past 9."*

Scoring: One point is allocated for each of the following three criteria:

© Z. Nasreddine MD

• Contour (1 pt.): the clock contour must be drawn (either a circle or a square). Only minor distortions are acceptable (e.g., slight imperfection on closing the circle). If the numbers are arranged in a circular manner but the contour is not drawn the contour is scored as incorrect.
• Numbers (1 pt.): all clock numbers must be present with no additional numbers. Numbers must be in the correct order, upright and placed in the approximate quadrants on the clock face. Roman numerals are acceptable. The numbers must be arranged in a circular manner (even if the contour is a square). All numbers must either be placed inside or outside the clock contour. If the subject places some numbers inside the clock contour and some outside the clock contour, (s)he does not receive a point for Numbers.
• Hands (1 pt.): there must be two hands jointly indicating the correct time. The hour hand must be clearly shorter than the minute hand. Hands must be centered within the clock face with their junction close to the clock center.

4. <u>Naming:</u>

<u>Administration:</u> Beginning on the left, the examiner points to each figure and says: *"Tell me the name of this animal."*

<u>Scoring:</u> One point is given for each of the following responses: (1) snake (or a type of snake like Boa or Cobra) (2) elephant (3) crocodile or alligator.

5. <u>Memory:</u>

<u>Administration:</u> The examiner reads a list of five words at a rate of one per second, giving the following instructions*: "This is a memory test. I am going to read a list of words that you will have to remember now and later on. Listen carefully. When I am through, tell me as many words as you can remember. It doesn't matter in what order you say them."* The examiner marks a check in the allocated space for each word the subject produces on this first trial. The examiner may not correct the subject if (s)he recalls a deformed word or a word that sounds like the target word. When the subject indicates that (s)he has finished (has recalled all words), or can recall no more words, the examiner reads the list a second time with the following instructions: *"I am going to read the same list for a second time. Try to remember and tell me as many words as you can, including words you said the first time."* The examiner puts a check in the allocated space for each word the subject recalls on the second trial. At the end of the second trial, the examiner informs the subject that (s)he will be asked to recall these words again by saying: *"I will ask you to recall those words again at the end of the test."*

<u>Scoring:</u> No points are given for Trials One and Two.

6. <u>Attention:</u>

<u>Forward Digit Span: Administration:</u> The examiner gives the following instructions: "*I am going to say some numbers and when I am through, repeat them to me exactly as I said them.*" The examiner reads the five number sequence at a rate of one digit per second.

<u>Backward Digit Span: Administration:</u> The examiner gives the following instructions: "*Now I am going to say some more numbers, but when I am through you must repeat them to me in the <u>backward</u> order.*" The examiner reads the three number sequence at a

© Z. Nasreddine MD

rate of one digit per second. If the subject repeats the sequence in the forward order, the examiner may not ask the subject to repeat the sequence in backward order at this point.

Scoring: One point is allocated for each sequence correctly repeated (N.B.: the correct response for the backward trial is 7-4-2).

Vigilance: Administration: The examiner reads the list of letters at a rate of one per second, after giving the following instructions: "*I am going to read a sequence of letters. Every time I say the letter A, tap your hand once. If I say a different letter, do not tap your hand.*"

Scoring: One point is allocated if there is zero to one error (an error is a tap on a wrong letter or a failure to tap on letter A).

Serial 7s: Administration: The examiner gives the following instructions: *"Now, I will ask you to count by subtracting 7 from 70, and then, keep subtracting 7 from your answer until I tell you to stop."* The subject must perform a mental calculation, therefore, (s)he may not use his/her fingers nor a pencil and paper to execute the task. The examiner may not repeat the subject's answers. If the subject asks what her/his last given answer was or what number (s)he must subtract from his/her answer, the examiner responds by repeating the instructions if not already done so.

Scoring: This item is scored out of 3 points. Give no (0) points for no correct subtractions, 1 point for one correct subtraction, 2 points for two or three correct subtractions, and 3 points if the subject successfully makes four or five correct subtractions. Each subtraction is evaluated independently; that is, if the subject responds with an incorrect number but continues to correctly subtract 7 from it, each correct subtraction is counted. For example, a subject may respond "62 - 55 - 48 - 41 – 34" where the "62" is incorrect, but all subsequent numbers are subtracted correctly. This is one error and the task would be given a score of 3.

7. Sentence repetition:

Administration: The examiner gives the following instructions: *"I am going to read you a sentence. Repeat it after me, exactly as I say it* [pause]: ***The robber of the gray car was stopped by the police.****"* Following the response, say: *"Now I am going to read you another sentence. Repeat it after me, exactly as I say it* [pause]: ***The student went back to school without his books and pencils.****"*

Scoring: One point is allocated for each sentence correctly repeated. Repetitions must be exact. Be alert for omissions (e.g., omitting "his"), substitutions/additions (e.g., substituting "and pencils" for "and his pencils"), grammar errors/altering plurals (e.g. "went" for "goes"), etc.

8. Verbal fluency:

Administration: The examiner gives the following instructions: *"Now, I want you to tell me as many words as you can think of that begin with the letter S. I will tell you to stop after one minute. Proper nouns, numbers, and different forms of a verb are not permitted. Are you ready?* [Pause] [Time for 60 sec.] *Stop."* If the subject names two consecutive

© Z. Nasreddine MD

words that begin with another letter of the alphabet, the examiner repeats the target letter if the instructions have not yet been repeated.

Scoring: One point is allocated if the subject generates 11 words or more in 60 seconds. The examiner records the subject's responses in the margins or on the back of the test sheet.

9. Abstraction:

Administration: The examiner asks the subject to explain what each pair of words has in common, starting with the example: *"I will give you two words and I would like you to tell me to what category they belong to* [pause]: *an orange and a banana.*" If the subject responds correctly the examiner replies: *''Yes, both items are part of the category Fruits.''* If the subject answers in a concrete manner, the examiner gives one additional prompt: *"Tell me another category* to *which these items belong to."* If the subject does not give the appropriate response *(fruits)*, the examiner says: *"Yes, and they also both belong to the category Fruits."* No additional instructions or clarifications are given. After the practice trial, the examiner says: *"Now, a bed and a table."* Following the response, the examiner administers the second trial by saying: *"Now, a letter and a telephone."* A prompt (one for the entire abstraction section) may be given if none was used during the example.

Scoring: Only the last two pairs are scored. One point is given for each pair correctly answered. The following responses are acceptable:

- bed- table = furniture, pieces of furniture, furnishings
- letter- telephone = communication, means of communication, means of corresponding

The following responses are **not** acceptable:

- bed-table = flat, four legs, bedroom, made out of wood
- letter- telephone = alphabet, they have letters, they have numbers, news, messages

10. Delayed recall:

Administration: The examiner gives the following instructions: *"I read some words to you earlier, which I asked you to remember. Tell me as many of those words as you can remember."* The examiner makes a check mark (√) for each of the words correctly recalled spontaneously without any cues, in the allocated space.

Scoring: **One point is allocated for each word recalled freely without any cues.**

Memory index score (MIS):

Administration: Following the delayed free recall trial, the examiner provides a category (semantic) cue for each word the subject was unable to recall. Example: ''*I will give you some hints to see if it helps you remember the words, the first word was a body part*.'' If the subject is unable to recall the word with the category cue, the examiner provides him/her with a multiple choice cue. Example: *"Which of the following words do you think it was, HAND, SHOULDER or FACE?"* All non-recalled words are prompted in this manner. The examiner identifies the words the subject was able to recall with the help of a cue (category or multiple-choice) by placing a

© Z. Nasreddine MD

check mark (√) in the appropriate space. The cues for each word are presented below:

**Target Word**	**Category Cue**	**Multiple Choice**
HAND	body part	hand, shoulder, face (nose, leg)
NYLON	type of fabric	cotton, nylon, silk (velvet, denim)
PARK	public place	park, library, school (church, hospital)
CARROT	type of food	tomato, carrot, lettuce (cucumber, celery)
YELLOW	color	yellow, purple, green (red, blue)

* The words in parentheses are to be used if the subject mentions one or two of the multiple choice responses during the category cuing.

Scoring: To determine the MIS (which is a sub-score), the examiner attributes points according to the type of recall (see table below). The use of cues provides clinical information on the nature of the memory deficits. For memory deficits due to retrieval failures, performance can be improved with a cue. For memory deficits due to encoding failures, performance does not improve with a cue.

**MIS scoring**				**Total**
Number of words recalled spontaneously	…	multiplied by	3	…
Number of words recalled with a category cue	…	multiplied by	2	…
Number of words recalled with a multiple choice cue	…	multiplied by	1	…
	**Total MIS** (add all points)			**---/15**

11. Orientation:

Administration: The examiner gives the following instructions: "*Tell me today's date*." If the subject does not give a complete answer, the examiner prompts accordingly by saying: *"Tell me the [year, month, exact date, and day of the week]."* Then the examiner says: *"Now, tell me the name of this place, and which city it is in."*

Scoring: One point is allocated for each item correctly answered. The date and place (name of hospital, clinic, office) must be exact. No points are allocated if the subject makes an error of one day for the day and date.

TOTAL SCORE: Sum all subscores listed on the right-hand side. Add one point for subject who has 12 years or fewer of formal education, for a possible maximum of 30 points. A final total score of 26 and above is considered normal.

Please refer to the MoCA website at www.mocatest.org for more information on the MoCA.

MONTREAL COGNITIVE ASSESSMENT (MoCA®)

Version 8.2 English

Name:
Education:
Sex:
Date of birth :
DATE :

VISUOSPATIAL / EXECUTIVE

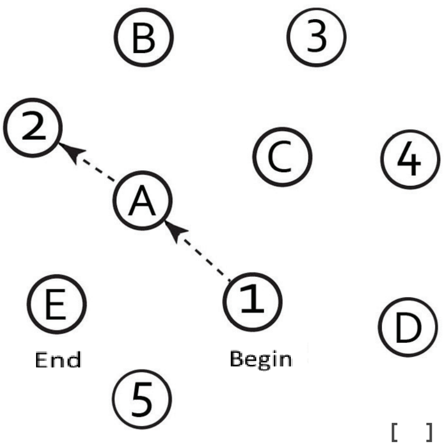

[ ]

Copy chair

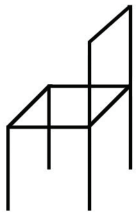

[ ]

Draw CLOCK (Ten past nine)
(3 points)

[ ] Contour [ ] Numbers [ ] Hands

POINTS

__/5

NAMING

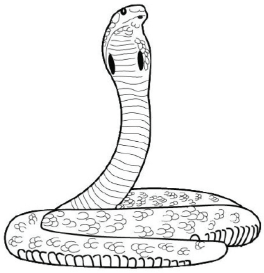

[ ]

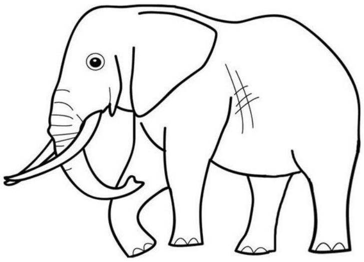

[ ]

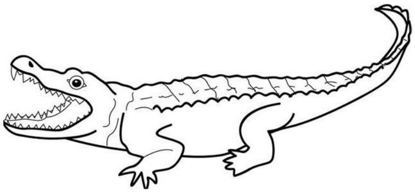

[ ]

__/3

MEMORY

Read list of words, subject must repeat them. Do 2 trials, even if 1st trial is successful. Do a recall after 5 minutes.

	HAND	NYLON	PARK	CARROT	YELLOW
1st TRIAL					
2nd TRIAL					

NO POINTS

ATTENTION

Read list of digits (1 digit / sec.). Subject has to repeat them in the forward order. [ ] 8 1 5 2 4
Subject has to repeat them in the backward order. [ ] 2 4 7

__/2

Read list of letters. The subject must tap with his hand at each letter A. No points if ≥ 2 errors.
[ ] F B A C M N A A J K L B A F A K D E A A A J A M O F A A B

__/1

Serial 7 subtraction starting at 70. [ ] 63 [ ] 56 [ ] 49 [ ] 42 [ ] 35
4 or 5 correct subtractions: **3 pts,** 2 or 3 correct: **2 pts,** 1 correct: **1 pt,** 0 correct: **0 pt**

__/3

LANGUAGE

Repeat: The robber of the gray car was stopped by the police. [ ]
The student went back to school without his books and pencils. [ ]

__/2

Language Fluency. Name maximum number of words in one minute that begin with the letter S. [ ] ______ (N ≥ 11 words)

__/1

ABSTRACTION

Similarity between e.g. banana - orange = fruit [ ] bed - table [ ] letter - telephone

__/2

DELAYED RECALL

(MIS)	Has to recall words WITH NO CUE	HAND [ ]	NYLON [ ]	PARK [ ]	CARROT [ ]	YELLOW [ ]	Points for UNCUED recall only
X3							
X2	Category cue						MIS = ____/15
X1	Multiple choice cue						

Memory Index Score (MIS)

__/5

ORIENTATION

[ ] Date [ ] Month [ ] Year [ ] Day [ ] Place [ ] City

__/6

© Z. Nasreddine MD

www.mocatest.org

Administered by: ______________________________

Training and Certification are required to ensure accuracy.

MIS: / 15
(Normal ≥ 26/30)
Add 1 point if ≤ 12 yr education

TOTAL __ /30
