# Supplementary material for: Electroacupuncture combined with cognitive rehabilitation outperforms cognitive rehabilitation alone in treating post-stroke cognitive impairment: a randomized controlled trial
Source: Front Neurol. 2025 Jan 29;16:1507475. doi: 10.3389/fneur.2025.1507475 (PMC11814160; doi:10.3389/fneur.2025.1507475)
Supplement: Supplementary file 2 [file Data_Sheet_2.pdf]

记忆力评估

听觉词语学习测验（华山版）（AVLT-H）

项目		即刻回忆 1	即刻回忆 2	即刻回忆 3	短延迟回忆 5min	长延迟回忆 15min
服饰类	大衣					
	长裤					
	头巾					
	手套					
职业类	司机					
	木工					
	士兵					
	律师					
花朵类	海棠					
	百合					
	腊梅					
	玉兰					
得分：		/12	/12	/12	/12	/12

结果与解释：若 50～59 岁，在长延迟回忆数<4 个，60～69 岁在长延迟回忆数<3 个，70 岁以上在长延迟回忆数<2 个，则可能存在轻度认知损伤。（适用人群：主要用于可能存在认知障碍的个体）
