# Supplementary material for: Electroacupuncture combined with cognitive rehabilitation outperforms cognitive rehabilitation alone in treating post-stroke cognitive impairment: a randomized controlled trial
Source: Front Neurol. 2025 Jan 29;16:1507475. doi: 10.3389/fneur.2025.1507475 (PMC11814160; doi:10.3389/fneur.2025.1507475)
Supplement: Supplementary file 3 [file Data_Sheet_3.pdf]

Multi-Ethnic Study of Atherosclerosis

MESA

Digit Span Test

Participant Id#:

Acrostic:

Tech ID#:

Date: ___ / ___ / ____
Month Day Year

DIGIT SPAN TEST - - *FORWARD*

- After saying the instructions administer the digit spans in order.
- Do not repeat a span once read.
- Administer both spans of the same length regardless of how the participant performs.
- Say the digits at a rate of 1 digit about every 1 sec.
- Use a monotonic voice; without inflections at the end
- Discontinue after failure on both trials of any item (e.g., 5a and 5b)

Examiner: *"I am going to say some numbers. Listen carefully, and when I am through say them right after me. For example, if I say 7-1-9, what would you say?"*

- If the participant responds correctly (7-1-9), say: *"That's right,"* and proceed to Item 1.
- If the participant fails the example, say: *"No, you would say 7-1-9. I said 7-1-9, so to say it forwards you would say 7-1-9. Now try these numbers. Remember, you are to say them forwards. 3-4-8."*
- Whether the participant succeeds or fails with the second example (3-4-8), proceed to Item 1. Give no help on this second example or any of the items that follow.

Scoring: Each span is scored '1' (Pass) or '0' (Fail). Only discontinue test when participant has failed both trials of the same span length (e.g., 5a and 5b)

Item		Digit Span	Pass	Fail
1	a.	1 - 7	○ 1	○ 0
	b.	6 - 3	○ 1	○ 0
2	a.	5 - 8 - 2	○ 1	○ 0
	b.	6 - 9 - 4	○ 1	○ 0
3	a.	6 - 4 - 3 - 9	○ 1	○ 0
	b.	7 - 2 - 8 - 6	○ 1	○ 0
4	a.	4 - 2 - 7 - 3 - 1	○ 1	○ 0
	b.	7 - 5 - 8 - 3 - 6	○ 1	○ 0
5	a.	6 - 1 - 9 - 4 - 7 - 3	○ 1	○ 0
	b.	3 - 9 - 2 - 4 - 8 - 7	○ 1	○ 0
6	a.	5 - 9 - 1 - 7 - 4 - 2 - 8	○ 1	○ 0
	b.	4 - 1 - 7 - 9 - 3 - 8 - 6	○ 1	○ 0
7	a.	5 - 8 - 1 - 9 - 2 - 6 - 4 - 7	○ 1	○ 0
	b.	3 - 8 - 2 - 9 - 5 - 1 - 7 - 4	○ 1	○ 0
8	a.	2 - 7 - 5 - 8 - 6 - 2 - 5 - 8 - 4	○ 1	○ 0
	b.	7 - 1 - 3 - 9 - 4 - 2 - 5 - 6 - 8	○ 1	○ 0

8038424313

DIGIT SPAN TEST - - *BACKWARD*

- Administer the digit spans in order.
- Do not repeat a span once read.
- Administer both spans of the same length regardless of how the participant performs.
- Say the digits at a rate of 1 digit about every 1 sec.
- Use a monotonic voice; without inflections at the end

Examiner: *"Now I am going to say some numbers, but this time when I stop I want you say them backwards. For example, if I say 7-1-9, what would you say?"*

- If the participant responds correctly (9-1-7), say: *"That's right,"* and proceed to Item 1.
- If the participant fails the example, say: *"No, you would say 9-1-7. I said 7-1-9, so to say it backwards you would say 9-1-7. Now try these numbers. Remember, you are to say them backwards. 3-4-8."*
- Whether the participant succeeds or fails with the second example (3-4-8), proceed to Item 1. Give no help on this second example or any of the items that follow.
- Discontinue after failure on both trials of any item (e.g., 5a and 5b)

Scoring: Each span is scored '1' (Pass) or '0' (Fail). Only discontinue test when participant has failed both trials of the same span length (e.g., 5a and 5b)

Item		Digit Span	Pass	Fail
1	a.	2 - 4	○ 1	○ 0
	b.	5 - 7	○ 1	○ 0
2	a.	6 - 2 - 9	○ 1	○ 0
	b.	4 - 1 - 5	○ 1	○ 0
3	a.	3 - 2 - 7 - 9	○ 1	○ 0
	b.	4 - 9 - 6 - 8	○ 1	○ 0
4	a.	1 - 5 - 2 - 8 - 6	○ 1	○ 0
	b.	6 - 1 - 8 - 4 - 3	○ 1	○ 0
5	a.	5 - 3 - 9 - 4 - 1 - 8	○ 1	○ 0
	b.	7 - 2 - 4 - 8 - 5 - 6	○ 1	○ 0
6	a.	8 - 1 - 2 - 9 - 3 - 6 - 5	○ 1	○ 0
	b.	4 - 7 - 3 - 9 - 1 - 2 - 8	○ 1	○ 0
7	a.	9 - 4 - 3 - 7 - 6 - 2 - 5 - 8	○ 1	○ 0
	b.	7 - 2 - 8 - 1 - 9 - 6 - 5 - 3	○ 1	○ 0
