# Supplementary material for: Electroacupuncture combined with cognitive rehabilitation outperforms cognitive rehabilitation alone in treating post-stroke cognitive impairment: a randomized controlled trial
Source: Front Neurol. 2025 Jan 29;16:1507475. doi: 10.3389/fneur.2025.1507475 (PMC11814160; doi:10.3389/fneur.2025.1507475)
Supplement: Supplementary file 4 [file Data_Sheet_4.pdf]

# 失语症筛查表

编号__________

姓名______ 性别_____ 年龄_____评价日期________________

| |
|---|
| 自发性言语： |

| 理 解 评 价 | | |
|---|---|---|
| I. 口语理解 | 正确 | 不正确 |
| **A. 一步指令：** | | |
| 1. “指给我哪是笔” | （ ） | （ ） |
| 2. “把勺子拿起来” | （ ） | （ ） |
| 3. “把杯子扣过来” | （ ） | （ ） |
| 4. “伸出你的手” | （ ） | （ ） |
| | 正答_____% | |
| **B. 两步指令：** | | |
| 1. “指给我哪是笔，然后拿起勺子”。 | （ ） | （ ） |
| 2. “拿起笔，把它放到杯子的右边”。 | （ ） | （ ） |
| 3. “先伸出两个手指，然后把勺子拿起来” | （ ） | （ ） |
| 4. “把肥皂递给我，然后用手指笔” | （ ） | （ ） |
| | 正答_____% | |
| **B. 三步指令：** | | |
| 1. “把笔放在被子里，递给我肥皂，再拿起勺子”。 | （ ） | （ ） |
| 2. “拿起勺子，把它放在肥皂的左边，再把被子扣过来”。 | （ ） | （ ） |
| 3. “指给我哪是灯，然后伸出两个手指，再闭上你的眼睛”。 | （ ） | （ ） |
| 4. “把笔放在肥皂和杯子之间，拿起勺子，再指你的鼻子”。 | （ ） | （ ） |
| | 正答_____% | |
| II. 书面语言理解 | | |
| **A. 单词理解** | | |
| 1. 杯子 | （ ） | （ ） |
| 2. 勺子 | （ ） | （ ） |
| 3. 肥皂 | （ ） | （ ） |
| 4. 笔 | （ ） | （ ） |
| | 正答_____% | |

| **B. 句子理解** | | |
|---|---|---|
| 1. 拿起勺子 | （ ） | （ ） |

| | | |
|---|---|---|
| 2. 伸出两个手指 | ( ) | ( ) |
| 3. 把勺子放在笔和肥皂之间 | ( ) | ( ) |
| 4. 把被子放在笔的左边 | ( ) | ( ) |
| | 正答_____% | |
| III. 手语的理解 | | |
| 1.杯子——示范用杯子喝水的动作,然后让病人指出摆在他面前用来喝水的物品。 | ( ) | ( ) |
| 2.笔——示范用笔写字的动作，然后让病人指出你所用来写字的物品。 | ( ) | ( ) |
| 3.肥皂——示范用肥皂洗手的动作,然后让病人指出你所用来洗手的物品。 | ( ) | ( ) |
| 4.饭勺——示范用勺吃饭的动作，然后让病人指出你所用来吃饭的物品。 | ( ) | ( ) |
| | 正答_____% | |

| 表 达 评 定 | | |
|---|---|---|
| I. 口语表达 | 正确 | 不正确 |
| **A. 命名** | | |
| 1. 杯子 | ( ) | ( ) |
| 2. 笔 | ( ) | ( ) |
| 3. 勺子 | ( ) | ( ) |
| 4. 肥皂 | ( ) | ( ) |
| | 正答_____% | |
| **B. 复述** | | |
| 1. 肥皂 | ( ) | ( ) |
| 2. 天安门广场 | ( ) | ( ) |
| 3. 请给我一支笔 | ( ) | ( ) |
| 4. 他穿过马路走进商店 | ( ) | ( ) |
| | 正答_____% | |
| **C. 描述** | | |
| 1. 肥皂 | ( ) | ( ) |
| 2. 勺子 | ( ) | ( ) |
| 3. 杯子 | ( ) | ( ) |
| 4. 笔 | ( ) | ( ) |
| | 正答_____% | |

| II. 书面语表达 | | |
|---|---|---|
| **A. 命名** | | |
| 1. 肥皂 | （ ） | （ ） |
| 2. 勺子 | （ ） | （ ） |
| 3. 杯子 | （ ） | （ ） |
| 4. 笔 | （ ） | （ ） |
| | 正答_____% | |
| **B. 描述** | | |
| 1. 肥皂 | （ ） | （ ） |
| 2. 勺子 | （ ） | （ ） |
| 3. 杯子 | （ ） | （ ） |
| 4. 笔 | （ ） | （ ） |
| | 正答_____% | |
| III. 手语表达 | | |
| 1. 勺子——指给我递给病人勺子，让病人示范它的应用。 | （ ） | （ ） |
| 2. 肥皂——与以上同样做法，让病人示范它的应用。 | （ ） | （ ） |
| 3. 笔——让病人示范如何使用它。 | （ ） | （ ） |
| 4. 杯子——让病人示范如何使用。 | （ ） | （ ） |
| | 正答_____% | |

评 价 结 果

| 理解 | | | 表达 | | |
|---|---|---|---|---|---|
| 口语 | 书面语 | 手语 | 口语 | 书写 | 手语 |
| 一步指令、二步、三步 | 词、句子 | | 命名、复述、描述 | 命名、描述 | |
| 100（ ） （ ） （ ） | （ ） （ ） | （ ） | （ ） （ ） （ ） | （ ） （ ） | （ ） |
| 75 （ ） （ ） （ ） | （ ） （ ） | （ ） | （ ） （ ） （ ） | （ ） （ ） | （ ） |
| 50 （ ） （ ） （ ） | （ ） （ ） | （ ） | （ ） （ ） （ ） | （ ） （ ） | （ ） |
| 25 （ ） （ ） （ ） | （ ） （ ） | （ ） | （ ） （ ） （ ） | （ ） （ ） | （ ） |
| 0 （ ） （ ） （ ） | （ ） （ ） | （ ） | （ ） （ ） （ ） | （ ） （ ） | （ ） |

初步诊断及治疗建议

| |
|---|
| |

检查者签名________
