# Supplementary material for: Electroacupuncture combined with cognitive rehabilitation outperforms cognitive rehabilitation alone in treating post-stroke cognitive impairment: a randomized controlled trial
Source: Front Neurol. 2025 Jan 29;16:1507475. doi: 10.3389/fneur.2025.1507475 (PMC11814160; doi:10.3389/fneur.2025.1507475)
Supplement: Supplementary file 5 [file Table_1.docx]

Table S1 Correlation analysis between MoCA and global properties of brain networks

|  | r | p |
| --- | --- | --- |
| Lp | 0.02181 | 0.4445 |
| Cp | 0.009269 | 0.6193 |
| Eg | 0.05759 | 0.2099 |

Table S2 Correlation analysis between AVLT-H and global properties of brain networks

|  | r | p |
| --- | --- | --- |
| Lp | 0.05483 | 0.1826 |
| Cp | 0.004235 | 0.7146 |
| Eloc | 0.05144 | 0.1971 |
| Eg | 0.007517 | 0.6259 |
